# Supplementary material for: Minimal impact of ZAP on lentiviral vector production and transduction efficiency
Source: Mol Ther Methods Clin Dev. 2021 Aug 28;23:147–57. doi: 10.1016/j.omtm.2021.08.008 (PMC8517000; doi:10.1016/j.omtm.2021.08.008)
Supplement: Document S1. Figures S1–S8 [file mmc1.pdf]

OMTM, Volume *23*

Supplemental information

Minimal impact of ZAP on lentiviral vector production and transduction efficiency

Helin Sertkaya, Laura Hidalgo, Mattia Ficarelli, Dorota Kmiec, Adrian W. Signell, Sadfer Ali, Hannah Parker, Harry Wilson, Stuart J.D. Neil, Michael H. Malim, Conrad A. Vink, and Chad M. Swanson

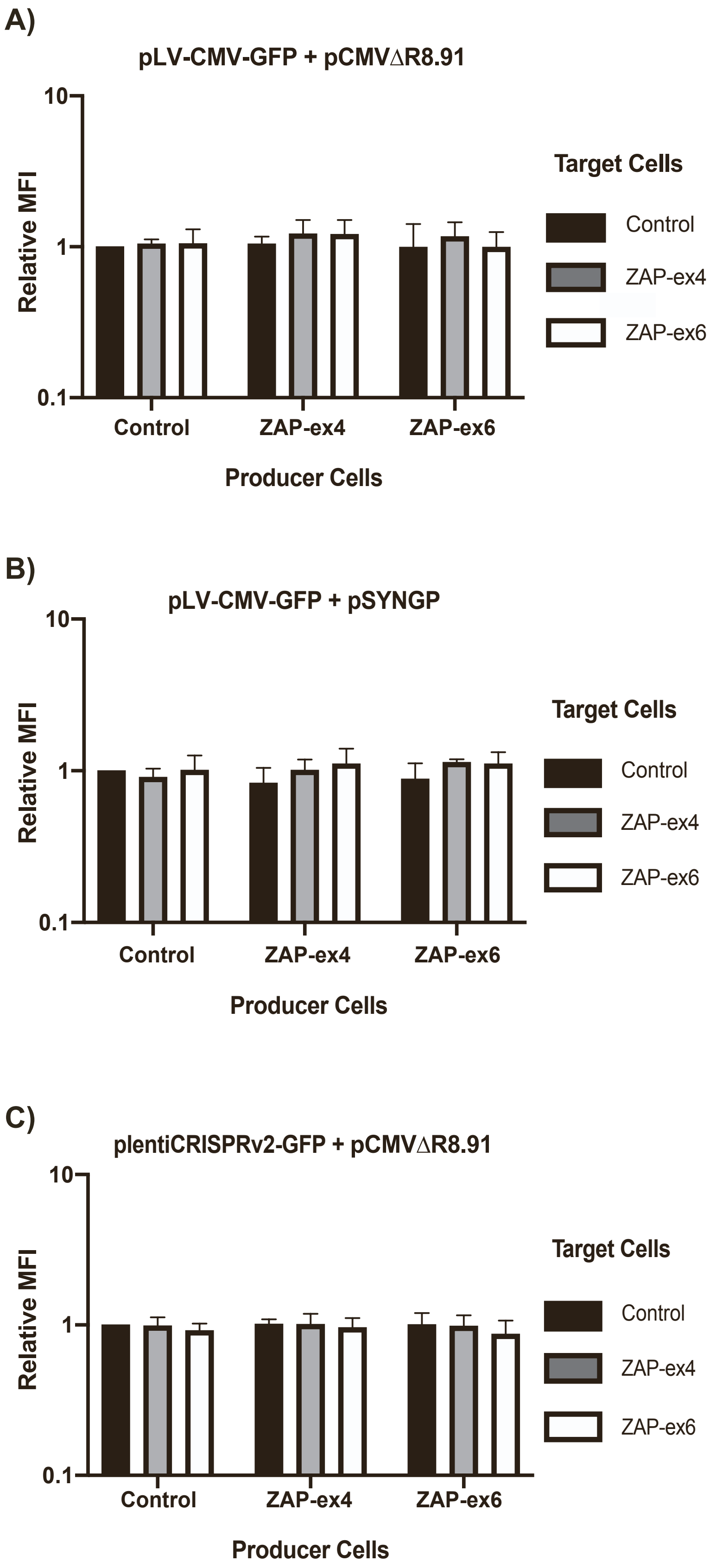

Figure S1. ZAP depletion does not affect transgene expression in a lentiviral vector.

Media from HEK293T producer cells (CRISPR control, ZAP-ex4 or ZAP-ex6) transfected with the indicated genome plasmid, packaging plasmid and pVSV-G was used to transduce target cells (HEK293T CRISPR control, ZAP-ex4 or ZAP-ex6 cells). MFI was determined by flow cytometry for GFP-positive target cells. The bar charts show the average values of three independent experiments. Data are shown as mean ± SD.

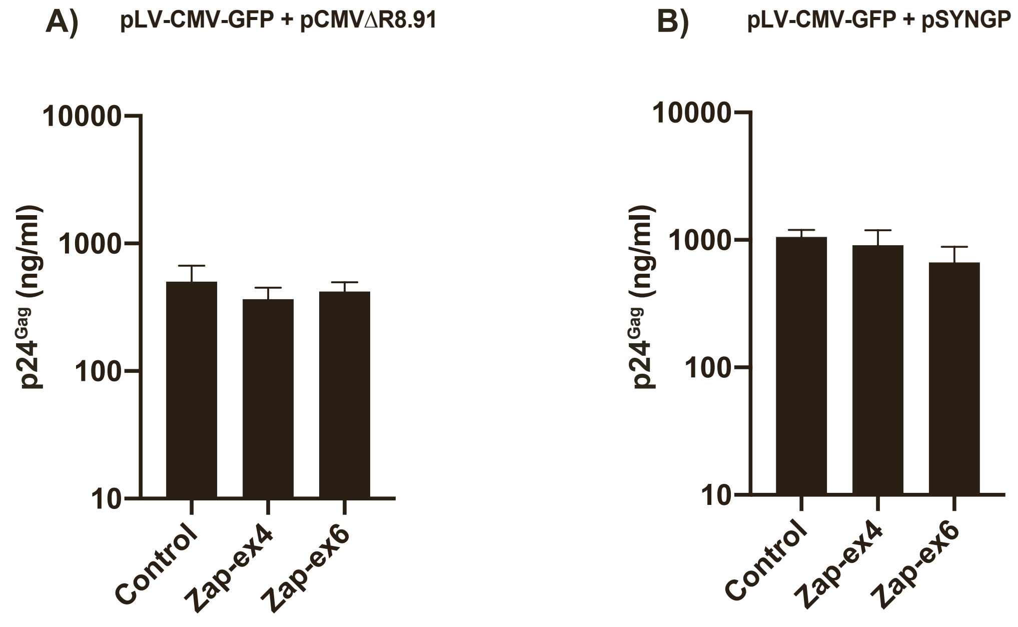

Figure S2. ZAP depletion does not affect $p24^{Gag}$ content in lentiviral vectors.

Media from HEK293T producer cells (CRISPR control, ZAP-ex4 or ZAP-ex6) transfected with the indicated genome and packaging plasmid and pVSV-G was used to quantify HIV-1 Capsid ($p24^{Gag}$) levels. The bar charts show the average values of three independent experiments. Data shown as mean ± SD.

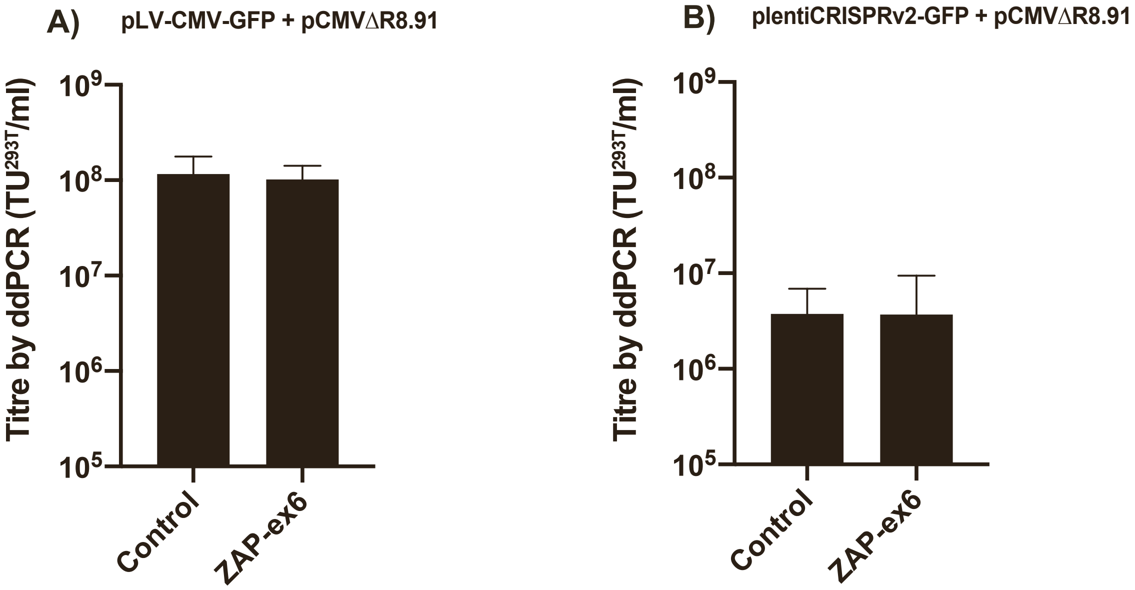

Figure S3. Endogenous ZAP in HEK293T cells does not restrict lentiviral vector titres measured by ddPCR

Media from HEK293T producer cells (CRISPR control or ZAP-ex6) transfected with the indicated genome plasmid, pCMVΔR8.91 and pVSV-G was used to transduce HEK293T cells. Infectious titres were determined in transduced cells by digital droplet PCR (ddPCR). Data shown as mean ± SD.

A)

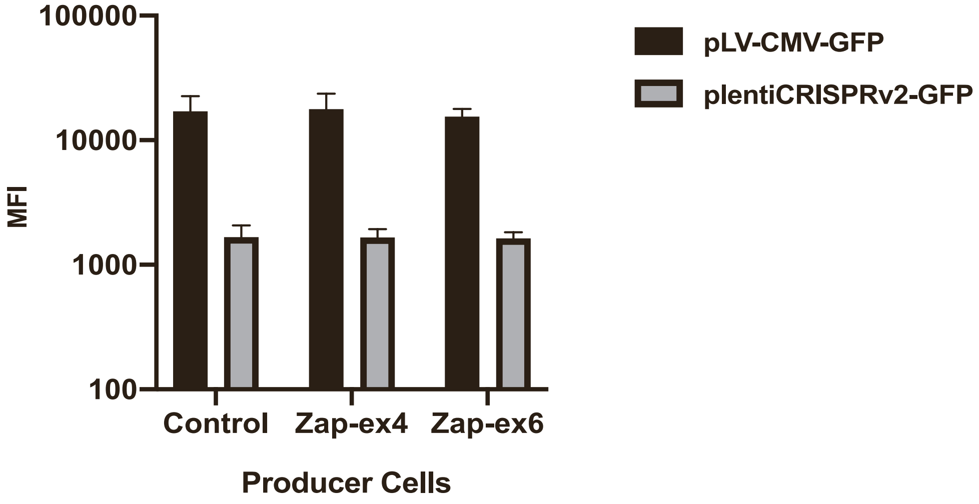

B)

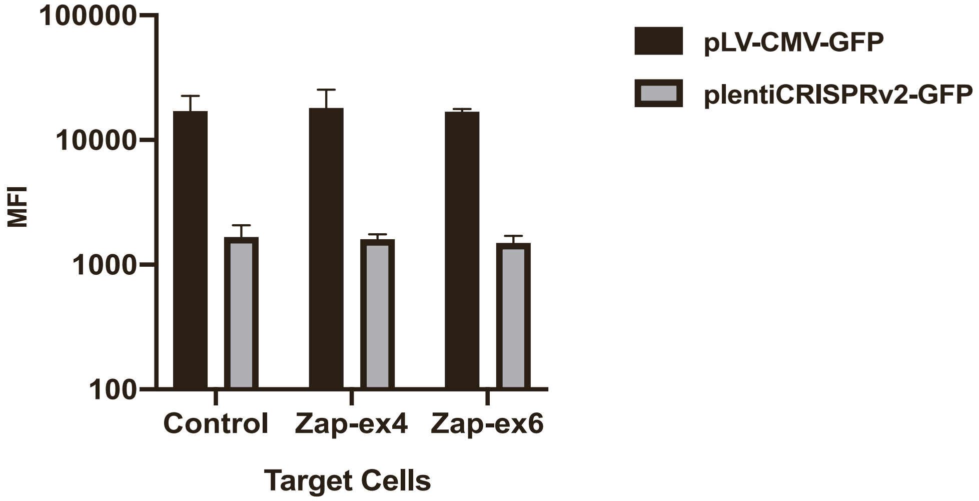

Figure S4. ZAP depletion does not affect the expression of transgenes under the control of CMV or EF-1a promoters.

(A) Media from HEK293T producer cells (CRISPR control, ZAP-ex4 or ZAP-ex6) transfected with pCMVΔR8.91, pVSV-G and the either pLV-CMV-GFP or plentiCRISPRv2-GFP, was used to transduce HEK293T CRISPR control target cells. **(B)** Media from HEK293T CRISPR control producer cells transfected with pCMVΔR8.91, pVSV-G and the either pLV-CMV-GFP or plentiCRISPRv2-GFP was used to transduce target cells (HEK293T CRISPR control, ZAP-ex4 or ZAP-ex6 cells). pLV-CMV-GFP contains the interval CMV promoter and plentiCRISPRv2-GFP contains the internal EF-1α promoter. MFI was determined by flow cytometry for GFP-positive target cells. The bar charts show the average values of six independent experiments. Data are shown as mean ± SD.

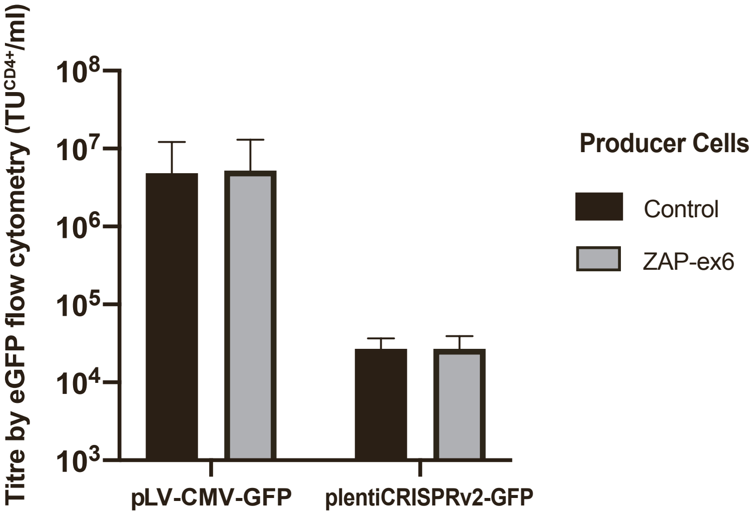

Figure S5. ZAP depletion in HEK293T producer cells does not increase vector titre in target primary human CD4+ T cells

Media from HEK293T CRISPR control and ZAP-ex6 producer cells transfected with pCMVΔR8.91, pVSV-G and pLV or plentiCRISPRv2-GFP was used to transduce target primary human CD4+ T cells. Infectious titres were determined by flow cytometry of GFP-positive target cells. The bar charts show the average values from four independent donors. Data are shown as mean ± SD.

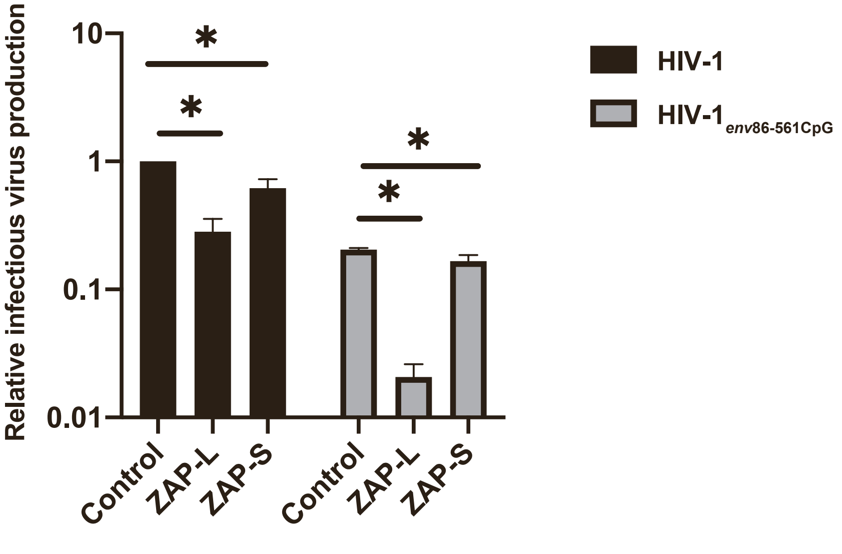

Figure S6. ZAP overexpression inhibits both wild type HIV-1 and HIV-$1_{env86\text{-}561CpG}$.

HEK293T cells were transfected with wild type pHIV-1 or pHIV-$1_{env86\text{-}561CpG}$ and either Control (pcDNA3), pZAP-L or pZAP-S. The culture supernatants were used to infect TZM-bl reporter cells to measure infectious virus production. The bar chart shows the average values of three independent experiments. Data are shown as mean ± SD, *$p<0.05$ as determined by an unpaired *t*-test.

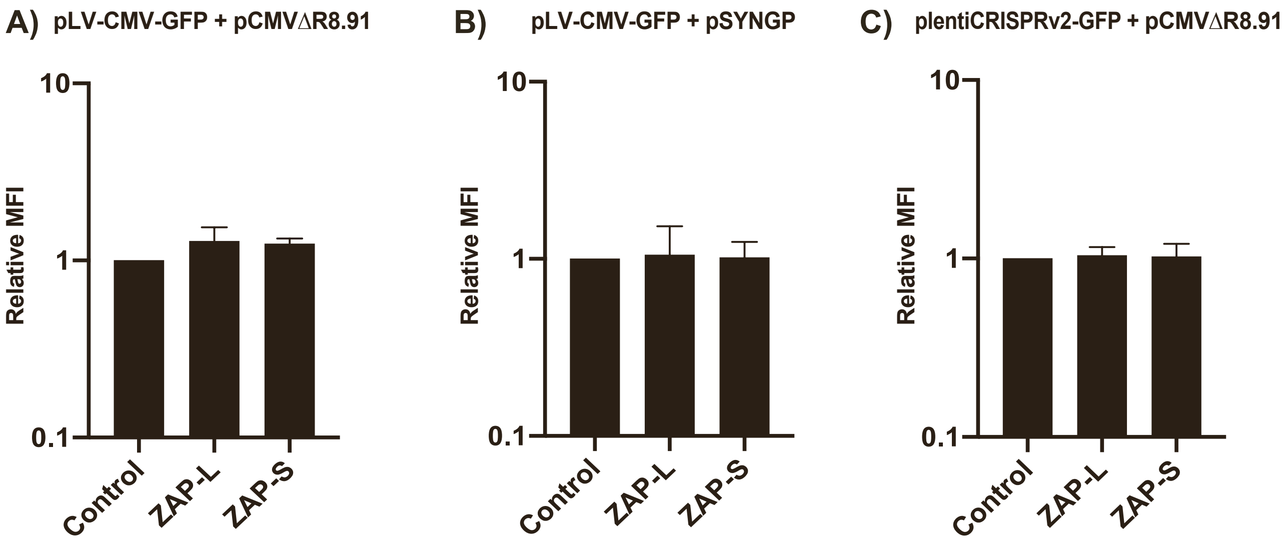

Figure S7. ZAP overexpression does not affect transgene expression in a lentiviral vector.

Media from HEK293T cells transfected with the indicated genome plasmid and packaging vector plus pVSV-G and either pcDNA4, pZAP-L or pZAP-S was used to transduce HEK293T target cells. MFI was determined by flow cytometry for GFP-positive target cells. The bar charts show the average values of three independent experiments. Data are shown as mean ± SD.

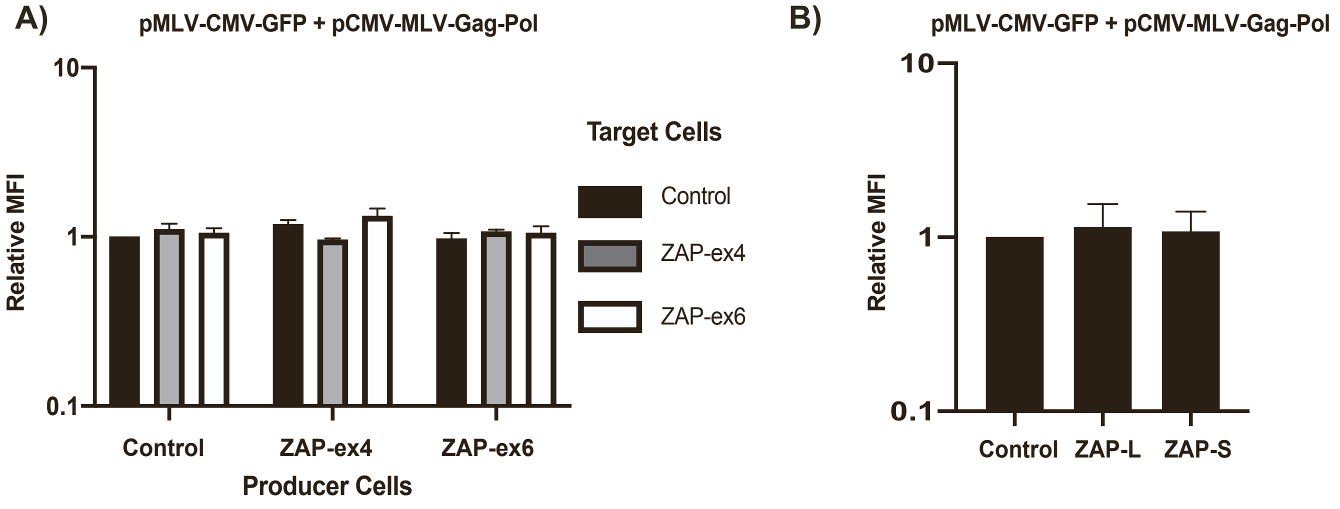

Figure S8. ZAP depletion or overexpression does not affect transgene expression in a gammaretroviral vector.

(A) Media from CRISPR control, ZAP-ex4 or ZAP-ex6 producer cells transfected with pMLV-CMV-GFP, pCMV-MLV-Gag-Pol and pVSV-G was used to transduce target cells (HEK293T CRISPR control, ZAP-ex4 or ZAP-ex6 cells). **(B)** Media from HEK293T cells transfected with pMLV-CMV-GFP, pCMV-MLV-Gag-Pol, pVSV-G and either pcDNA4, pZAP-L or pZAP-S was used to transduce HEK293T target cells. MFI was determined by flow cytometry for GFP-positive target cells.The bar charts show the average values of three independent experiments. Data are shown as mean ± SD.
