# Supplementary material for: Minimal impact of ZAP on lentiviral vector production and transduction efficiency
Source: Mol Ther Methods Clin Dev. 2021 Aug 28;23:147–57. doi: 10.1016/j.omtm.2021.08.008 (PMC8517000; doi:10.1016/j.omtm.2021.08.008)
Supplement: Document S2. Article plus supplemental information [file mmc2.pdf]

Molecular Therapy
Methods & Clinical Development
Original Article

Minimal impact of ZAP on lentiviral vector production and transduction efficiency

Helin Sertkaya,[1] Laura Hidalgo,[1] Mattia Ficarelli,[1] Dorota Kmiec,[1] Adrian W. Signell,[1] Sadfer Ali,[2] Hannah Parker,[1] Harry Wilson,[1] Stuart J.D. Neil,[1] Michael H. Malim,[1] Conrad A. Vink,[2] and Chad M. Swanson[1]

[1]Department of Infectious Diseases, King's College London, London SE1 9RT, UK; [2]Cell & Gene Therapy Platform, Medicinal Science and Technology, GSK, Stevenage SG1 2NY, UK

The antiviral protein ZAP binds CpG dinucleotides in viral RNA to inhibit replication. This has likely led to the CpG suppression observed in many RNA viruses, including retroviruses. Sequences added to retroviral vector genomes, such as internal promoters, transgenes, or regulatory elements, substantially increase CpG abundance. Because these CpGs could allow retroviral vector RNA to be targeted by ZAP, we analyzed whether it restricts vector production, transduction efficiency, and transgene expression. Surprisingly, even though CpG-high HIV-1 was efficiently inhibited by ZAP in HEK293T cells, depleting ZAP did not substantially increase lentiviral vector titer using several packaging and genome plasmids. ZAP overexpression also did not inhibit lentiviral vector titer. In addition, decreasing CpG abundance in a lentiviral vector genome did not increase its titer, and a gammaretroviral vector derived from murine leukemia virus was not substantially restricted by ZAP. Overall, we show that the increased CpG abundance in retroviral vectors relative to the wild-type retroviruses they are derived from does not intrinsically sensitize them to ZAP. Further understanding of how ZAP specifically targets transcripts to inhibit their expression may allow the development of CpG sequence contexts that efficiently recruit or evade this antiviral system.

INTRODUCTION

Retroviral vectors are a key tool for gene delivery for a wide variety of therapeutic treatments including inherited immune or metabolic disorders and chimeric antigen receptor T cell (CAR-T cell) anticancer therapies.[1] They are most often based on either the gammaretrovirus murine leukemia virus (MLV) or the lentivirus human immunodeficiency virus type 1 (HIV-1). For example, Strimvelis is an MLV-based gammaretroviral vector therapy for severe combined immunodeficiency caused by adenosine deaminase deficiency (ADA-SCID)[2] and Kymriah uses an HIV-1-based lentiviral vector to deliver an autologous CAR-T cell immunotherapy for acute lymphoblastic leukemia.[3]

Identifying cellular proteins that promote or inhibit retroviral vector production, transduction efficiency, and transgene expression is essential to optimize producer cells and identify transduction enhancers to promote efficient transgene expression in a wide range of target cell types. Retrovirus replication can be restricted by several components of the cell-autonomous innate immune system including APOBEC3 proteins, TRIM5α, tetherin/BST2, SAMHD1, IFITM proteins, MX2, and ZAP.[4] Retroviral vectors can also potentially be inhibited by these proteins.[5] In producer cells, APOBEC3 proteins and tetherin could potentially restrict vector production, although these proteins are not expressed in HEK293T cells.[6–8] In at least some types of target cells, IFITM proteins, TRIM5α, SAMHD1, and MX2 inhibit retroviral vector transduction by targeting entry, reverse transcription, or nuclear import.[9–16] Highlighting the importance of antiviral proteins in determining the susceptibility of target cells for transduction, cyclosporin H was recently identified to promote lentiviral vector transduction of human hematopoietic stem cells by inhibiting IFITM3 expression.[17]

ZAP is an antiviral protein that is broadly expressed in human cells and restricts a diverse range of viruses.[18] It was initially identified based on its ability to inhibit MLV gene expression and targets viral RNA for degradation and/or inhibits its translation.[19–22] There are several ZAP isoforms, all of which contain four N-terminal zinc finger motifs that bind RNA.[23–25] The most antiviral isoform, ZAP long (ZAP-L), also has a C-terminal catalytically inactive PARP domain and S-farnesylation motif.[24,26,27] ZAP has no known enzymatic activity and must interact with other cellular proteins such as the 3′–5′ exosome, TRIM25, KHNYN, or OAS3/RNase L to inhibit viral gene expression.[21,28–32]

Because ZAP is expressed in most cell types, it could reduce infectious vector yield from producer cells and transgene expression in target cells. ZAP directly binds CpG dinucleotides in RNA through its second zinc finger motif (Zn2), and this is believed to be required for it to target viral transcripts,[30,33–37] although UpA dinucleotides have also been implicated.[31,32,38] CpG dinucleotides are highly suppressed in retroviruses, including HIV-1, and increasing their frequency inhibits viral replication.[30,33,36,39–42] Whereas it was initially hypothesized

Received 8 December 2020; accepted 24 August 2021;
https://doi.org/10.1016/j.omtm.2021.08.008.

Correspondence: Chad M. Swanson, Department of Infectious Diseases, King's College London, London SE1 9RT, UK.
E-mail: chad.swanson@kcl.ac.uk

This is an open access article under the CC BY license (http://creativecommons.org/licenses/by/4.0/).

that CpGs are suppressed in HIV-1 due to the consequences of DNA methylation at these sites, it has recently been reported that one of the major evolutionary forces for the low frequency of CpGs in this virus is likely due to ZAP binding them in the viral RNA.[33,39,40,42,43] However, there is currently limited understanding of how ZAP specifically targets viral RNAs, the role of the sequence surrounding the CpG remains unclear for ZAP antiviral activity, and the presence of high local CpG frequency in viral RNA is not always sufficient for ZAP to restrict replication.[32–37] For example, ZAP targets HIV-1 most potently when CpGs are introduced in a specific region of *env* due to unknown factors.[33,36,37] In the process of engineering lentiviral vector systems from HIV-1, CpGs were introduced into several regions of the vector genome[44] that could sensitize the genomic RNA to ZAP in the producer or target cells. Importantly, the internal promoter and transgene often have different nucleotide and codon biases compared to HIV-1, which increases CpG abundance. The woodchuck hepatitis virus post-transcriptional regulatory element (WPRE) added to many lentiviral vectors[45] also contains CpGs that could allow ZAP to target the vector RNA.

Of note, if ZAP did target CpGs in retroviral vector RNA, then this would be independent of any effects the CpGs in the integrated DNA had on the transcriptional regulation of the transgene, such as position effect variegation.[46] This occurs when identical genes at different positions in a genome have differential expression. For example, retroviral vector integration into euchromatin leads to strong expression of the transgene, whereas integration into heterochromatin results in weak or no expression. One of the mechanisms leading to position effect variegation could be CpG DNA methylation leading to transgene silencing, and insulator elements have been used to avoid this.[47] However, CpG DNA methylation and ZAP-mediated repression are separate mechanisms for inhibiting retroviral vector transgene expression because CpGs in the integrated vector DNA are potentially methylated, whereas ZAP post-transcriptionally targets CpGs in RNA.

To determine whether ZAP restricts lentiviral vector production or transgene expression, we analyzed whether ZAP depletion or overexpression modulates vector titer. Overall, whereas ZAP depletion increased the production of infectious HIV-1 with increased CpG abundance in *env*, the titer of lentiviral vectors with two different internal promoters and transgenes was not significantly affected. ZAP overexpression in producer cells also did not inhibit the lentiviral vector titer. Increasing CpG abundance in the Gag-Pol packaging construct did not sensitize lentiviral vector production to ZAP, and decreasing CpG abundance in a lentiviral vector genome did not increase its titer. Furthermore, depleting ZAP in target cells did not affect vector titer or transgene expression. We also found that ZAP did not substantially inhibit the titer of a gammaretroviral vector. Overall, these results show that increasing the CpG frequency in retroviral vectors far above that found in the wild-type viruses does not intrinsically sensitize the vector to ZAP. Although this reduces a potential concern for optimizing lentiviral vector production and transduction efficiency, other sequences not tested could mediate restriction, and further understanding of how ZAP mediates its antiviral activity is required to produce sequence contexts that will reliably avoid inhibition by this antiviral protein.

RESULTS

Endogenous ZAP in HEK293T cells does not substantially inhibit lentiviral vector production, transduction efficiency, or gene expression

The role of ZAP for inhibiting retroviral vector production or gene expression has predominately been analyzed by ZAP overexpression in HEK293T cells. These studies found that ZAP overexpression inhibited luciferase expression in the context of the MLV 3′ long terminal repeat (LTR) but not from an HIV-1 LTR-luciferase vector.[19,24,25] In contrast, a lentiviral vector genome encoding Cas9 and a puromycin-resistance gene (puromycin-N-acetyltransferase) were shown to be targeted by ZAP in a CRISPR-Cas9-based depletion screen for interferon-induced genes in monocyte-like THP-1 cells.[48] Therefore, we sought to determine whether endogenous levels of ZAP in HEK293T cells, which are the most commonly used cell line for retroviral vector production,[49] could inhibit lentiviral vector titer.

We first characterized cell lines in which ZAP was depleted by CRISPR-Cas9-mediated genome engineering. In HEK293T cells with a guide RNA targeting exon 4 (ZAP-ex4), ZAP-L was depleted by ∼50% and ZAP short isoform (ZAP-S) was depleted by >80% compared to the control CRISPR cells (expressing a guide RNA targeting firefly luciferase; Figure 2B). In HEK293T cells containing the guide RNA targeting exon 6 (ZAP-ex6), both isoforms were depleted by >90%. When these cells were transfected with either wild-type HIV-1 (Figure 1A; 83 CpGs in the genomic RNA, 9.0 CpGs/kb; Table 1) or a ZAP-sensitive HIV-1 virus (HIV-$1_{env86-561}$CpG containing 36 CpGs introduced into *env*, 119 CpGs total, 13.0 CpGs/kb),[30,36] HIV-$1_{env86-561}$CpG infectivity, as measured in TZM-bl target cells, was increased to levels similar to wild-type HIV-1 (Figure 2A). HIV-$1_{env86-561}$CpG Gag expression and virion production were also not inhibited in the ZAP CRISPR cells (Figure 2B). These results validated these cells for analyzing the effect of ZAP on retroviral vector titer.

To investigate the effect of ZAP in producer cells, lentiviral vectors expressing GFP were produced in either control or ZAP CRISPR HEK293T cells. The vectors were made with pLV-CMV-GFP[50] (Figure 1B), which contains a CMV enhancer/promoter (34 CpGs), GFP open reading frame (60 CpGs), and WPRE (37 CpGs). It has a total of 182 CpGs in the genomic RNA (46.0 CpGs/kb; Table 1). The packaging plasmid (p) was pCMVΔR8.91,[51] which contains 86 CpGs in the unspliced RNA (13.5 CpGs/kb; Figure 1D; Table 1). Interestingly, despite the lentiviral vector genome having a 5-fold greater CpG/kb frequency than HIV-1, there was only a small (∼2-fold), non-significant increase in the vector titer in ZAP CRISPR cells (Figure 3A). Consistent with this effect, depleting ZAP did not increase Gag expression or virion production in the producer cells when analyzed by western blotting or p24 ELISA

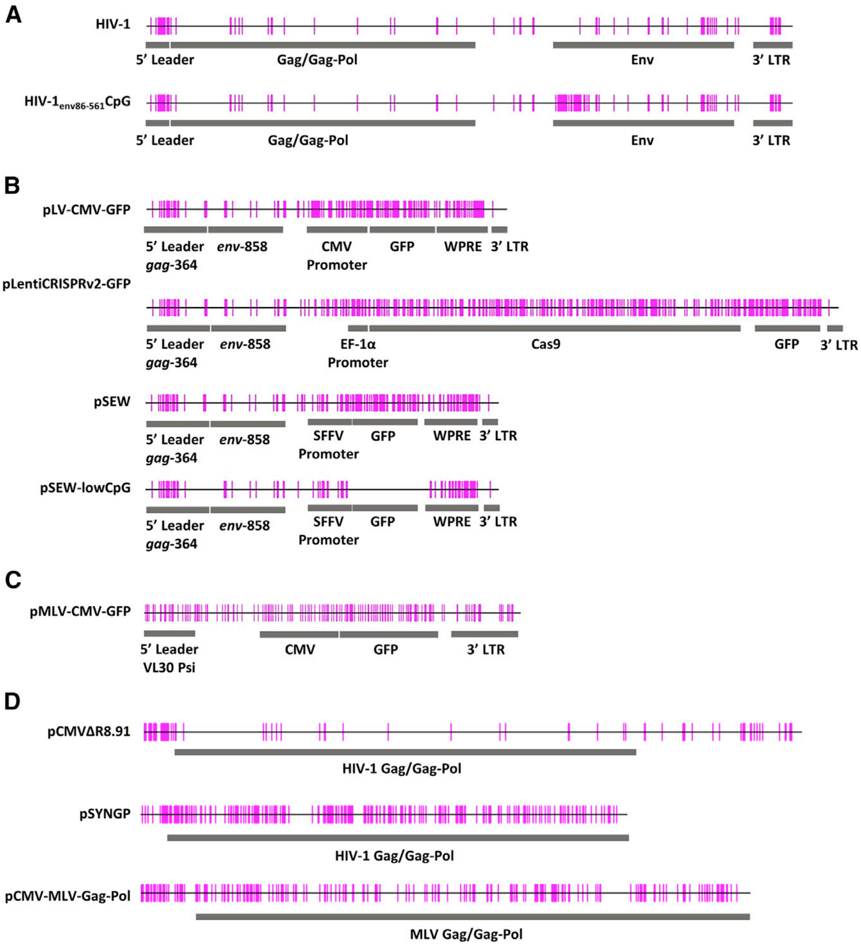

Figure 1. Schematic of CpG dinucleotides in retroviral vector genomes

DNA sequences were analyzed for CpG sites using Methyl Primer Express (Applied Biosystems). Genomes within each panel are to scale. (A) CpG dinucleotides annotated for wild-type HIV-1 and HIV-$1_{env86-561}$CpG genomic RNAs. (B) CpG dinucleotides annotated for lentiviral vector genomes pLV-CMV-GFP, plentiCRISPRv.2-GFP, pHR'SIN-cPPT-SEW (pSEW), and pSEW-lowCpG genomic RNAs. (C) CpG dinucleotides annotated for MLV-CMV-GFP retroviral vector genomic RNA. (D) CpG dinucleotides annotated for the packaging vectors pCMVΔR8.91, pSYNGP, and pCMV-MLV-Gag-Pol.

(Figure 3B; Figure S2A). ZAP depletion also did not alter the titer of LV-CMV-GFP when it was quantified by digital droplet PCR (ddPCR) (Figure S3A).

We also analyzed whether ZAP depletion in target cells increased transduction efficiency. When the ZAP CRISPR cells were used as target cells, similar titers were observed compared to control CRISPR cells (Figure 3A). ZAP depletion also did not alter the mean fluorescence intensity (MFI) for GFP in the target cells (Figure S1A). This indicates that ZAP expression in the producer or target cell does not substantially inhibit pLV-CMV-GFP vector titer or transgene expression.

Rev-independent, codon-modified Gag-Pol constructs have been produced to potentially increase the safety profile of lentiviral vectors.[52,53] However, vectors produced using these plasmids often have lower titers than vectors produced with Rev-dependent Gag-Pol packaging constructs.[49,52,53] Codon modification often introduces CpGs into the mRNA through synonymous mutations, and it is possible that these inhibit vector production in a ZAP-dependent manner. The Rev-independent Gag-Pol vector pSYNGP[53] contains 237 CpGs in the mRNA (50.1 CpGs/kb; Figure 1D; Table 1), and we tested whether ZAP depletion increases vector titer when it is used as the packaging construct. Despite the ~4-fold increase in CpG frequency in the *gag-pol* RNA from pSYNGP compared to pCMVΔR8.91, ZAP did not restrict lentiviral vector titer or transgene expression when pSYNGP was used with pLV-CMV-GFP (Figure 3C; Figure S1B). Gag expression and virion production from this vector were also not restricted by ZAP (Figure 3D; Figure S2B).

We then tested whether ZAP restricted a lentiviral vector that contained a different promoter (the elongation factor 1a short promoter) and a transgene containing a large number of CpGs, Cas9 (Figure 1B). For these experiments, we used a modified version of plentiCRISPRv.2[54] in which the puromycin selection marker was replaced with GFP to allow rapid analysis of vector titer. The genomic RNA for this vector has 399 CpGs (52.3 CpGs/kb; Table 1). plentiCRISPRv.2-GFP vectors were produced in control CRISPR or ZAP CRISPR HEK293T cells with the pCMVΔR8.91 packaging plasmid, and titers were measured in both control and ZAP CRISPR target cells. Despite the large number of CpGs in this vector, ZAP did not inhibit its titer or transgene expression (Figures 3E and 3F; Figure S1C; Figure S3B). Of note, even though plentiCRISPRv.2-GFP has a lower titer and expresses GFP at a lower level than pLV-CMV-GFP, this does not affect whether ZAP inhibits its transduction efficiency or transgene expression (Figures 3A and 3E; Figures S3A and S3B; Figures S4A and S4B).

To determine if ZAP depletion in producer cells affected the vector titer when primary cells were used as target cells, primary activated human CD4$^+$ T cells from four donors were transduced with pLV-CMV-GFP or plentiCRISPRv.2-GFP vectors produced in either control or ZAP CRISPR HEK293T cells (Figure S5). The vectors had similar titers when produced in each cell line, indicating that ZAP

Table 1. CpG frequency in HIV-1 and retroviral vectors

Construct	CpG dinucleotides in RNA	RNA length (nucleotides)	CpG dinucleotides/ kilobase
HIV-1	83	9,173	9.0
HIV-1$_{env86-561}$CpG	119	9,173	13.0
pLV-CMV-GFP	182	3,956	46.0
pCMVΔR8.91	86	6,352	13.5
pSYNGP	237	4,726	50.1
plentiCRISPRv.2-GFP	399	7,631	52.3
pHR'SIN-cPPT-SEW	180	3,920	45.9
pHR'SIN-cPPT-SEW-lowCpG	94	3,934	23.9
pMLV-CMV-GFP	171	2,755	62.1
pCMV-MLV-Gag-Pol	230	5,924	38.8

does not restrict vector titer in the context of a clinically relevant target cell type.

ZAP overexpression does not inhibit lentiviral vector production or transduction efficiency

To ascertain whether higher levels of ZAP inhibited lentiviral vector titer, ZAP-L or ZAP-S were overexpressed in HEK293T producer cells. Of note, we[36,37] and others[55] have previously shown that ZAP overexpression inhibits wild-type HIV-1 and HIV-1 with CpGs introduced in several different places in the genome, although ZAP-L is more potent than ZAP-S (Figure S6). Neither ZAP-L nor ZAP-S inhibited vector titer in HEK293T cells or transgene expression when pLV-CMV-GFP was the genome plasmid, and pCMVRΔ8.91 was the packaging plasmid, even though both ZAP-L and ZAP-S were expressed at high levels (Figure 4A; Figure S7A). Furthermore, neither protein inhibited Gag expression nor virion production. Similar results were observed when pSYNGP was the packaging construct, or plentiCRISPRv.2-GFP was the vector genome (Figures 4B and 4C; Figures S7B and S7C).

Decreasing CpG abundance in a lentiviral vector does not increase its titer

We also analyzed the effect of decreasing CpG abundance in the lentiviral vector genome. The number of CpGs in the genome vector pHR'SIN-cPPT-SEW[56] (pSEW; Figure 1B) was decreased from 180 (45.7 CpGs/kb) to 94 (pSEW-lowCpG; 23.9 CpGs/kb; Table 1) by eliminating CpGs in regions that do not contain known regulatory RNA structures or nucleic acid elements. Surprisingly, removing these CpGs led to a small but non-significant decrease in the vector titer in Jurkat target cells (Figure 5A). There was no effect on GFP expression in the transduced target cells (Figure 5B). Whereas altering the CpG abundance could affect RNA expression,[57] we observed no substantial change in intracellular or virion-associated genomic RNA abundance and a small increase in GFP expression from pSEW-lowCpG in producer cells (Figures 5C–5E). Therefore, decreasing CpG abundance does not enhance lentiviral vector production or transduction efficiency.

ZAP does not substantially inhibit gammaretroviral vector production, transduction efficiency, or transgene expression

Because ZAP has been shown to inhibit MLV,[19,25,30,58] we tested whether it inhibits an MLV-based gammaretroviral vector expressing GFP (pMLV-CMV-GFP; Figure 1C),[59] which has 171 CpGs (62.1 CpGs/kb; Table 1). pCMV-MLV-Gag-Pol, which has 230 CpGs in the *gag-pol* mRNA (38.8 CpGs/kb; Figure 1D; Table 1), was used as the packaging vector. Of note, the wild-type MLV genomic RNA has 36 CpGs/kb.[36] Similar to the results we observed for pLV-CMV-GFP (Figure 3A), depleting ZAP in producer cells led to a small (~2-fold), non-significant increase in the pMLV-CMV-GFP titer (Figure 6A). Depleting ZAP did not substantially affect Gag expression or virion production (Figure 6B), and there was no effect on vector titer or GFP expression when ZAP was depleted in target cells (Figure 6A; Figure S8A). ZAP-L overexpression in producer cells led to a small (~2-fold), non-significant decrease in the vector titer and no substantial change in Gag expression, virion production, or transgene expression (Figures 6C and 6D; Figure S8B). ZAP-S overexpression did not affect Gag expression, virion production, titer,

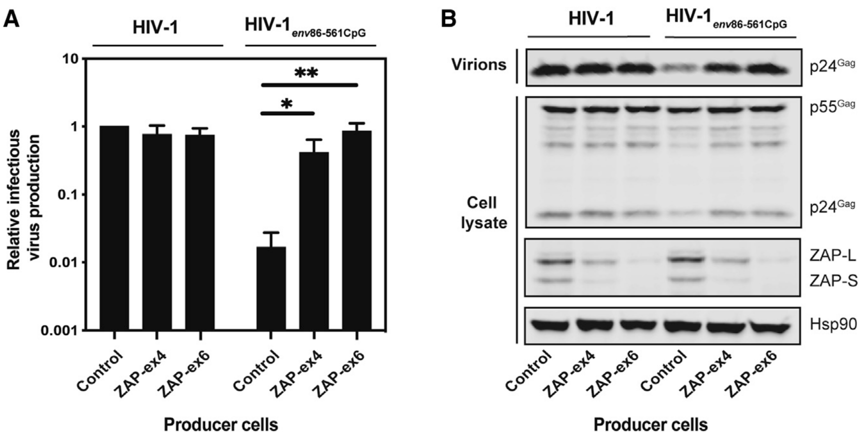

Figure 2. ZAP restricts HIV-1 with CpG dinucleotides introduced in *env* nucleotides (nt) 86–561

(A) HEK293T CRISPR control, ZAP-ex4, and ZAP-ex6 cells were transfected with wild-type pHIV-1 or pHIV-1$_{env86-561CpG}$. The culture supernatants were used to infect TZM-bl reporter cells to measure infectious virus production. The bar chart shows the average values of three independent experiments. Data are shown as mean ± SD, $*p < 0.05$, as determined by an unpaired t test. (B) Intracellular HIV-1 Gag and ZAP expression were determined by western blotting. Virus production was determined by western blot of the producer cell supernatant.

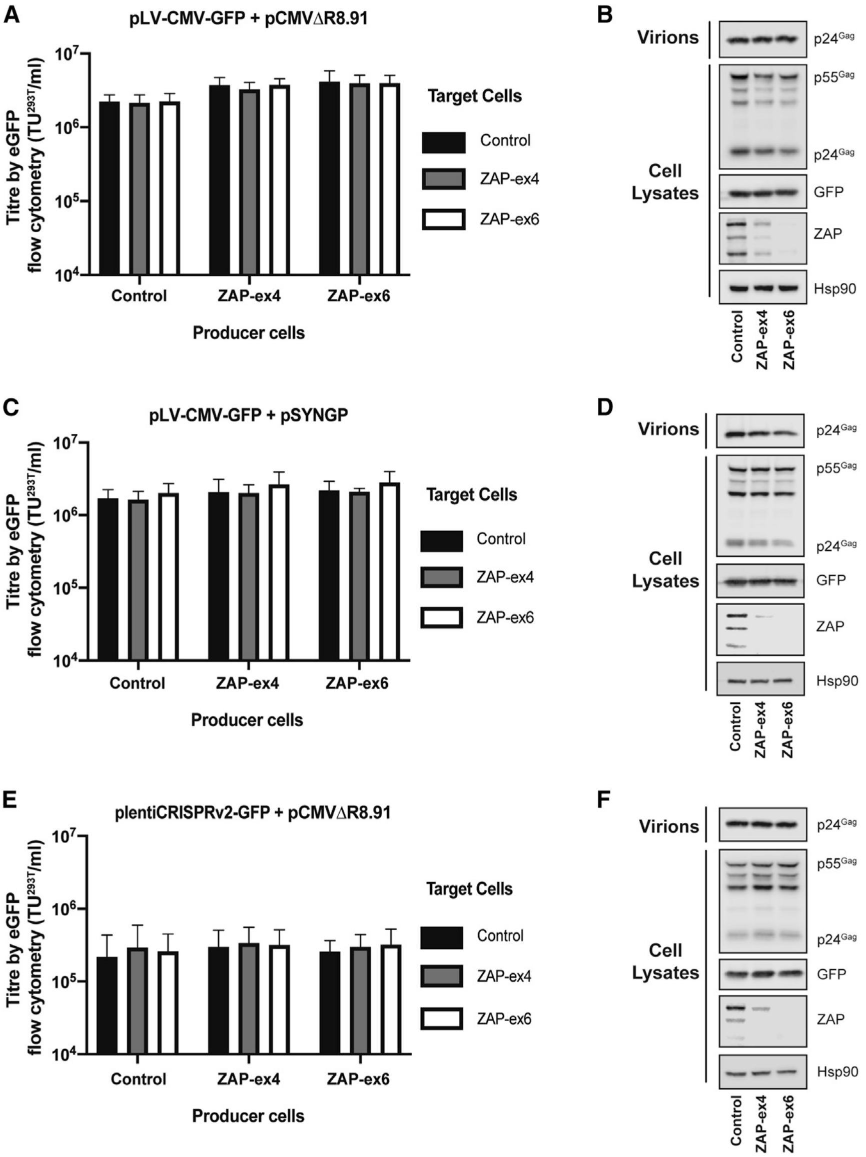

Figure 3. Endogenous ZAP in HEK293T cells does not restrict lentiviral vector titers

HEK293T producer cells (CRISPR control, ZAP-ex4, or ZAP-ex6) were transfected with the indicated genome plasmid, packaging plasmid, and pVSV-G. (A, C, and E) Infectious titers were determined in transduced target cells (HEK293T CRISPR control, ZAP-ex4, or ZAP-ex6 cells) by flow cytometry for GFP-positive cells. The bar charts show the average values of three independent experiments. Data are shown as mean ± SD. (B, D, and F) Intracellular HIV-1 Gag expression from the packaging vector, GFP expression from the vector genome, and HSP90 and ZAP expression were determined by western blots of the producer cell lysates. Virus production was determined by western blots of the producer cell supernatant.

or transgene expression (Figures 6C and 6D; Figure S8B). Together, these results show that ZAP does not appear to substantially inhibit a gammaretroviral vector derived from MLV.

DISCUSSION

Identifying antiviral proteins that restrict retroviral vectors is critical to optimize vector production and gene transfer efficiency. If these proteins are expressed in producer cells, then they could inhibit vector production, whereas in target cells, they could inhibit transduction efficiency or transgene expression. ZAP is broadly expressed and could potentially bind retroviral vector RNA in both producer and target cells. Wild-type HIV-1 is highly suppressed for CpG dinucleotides, which allows it to evade restriction by ZAP.[30,33,36,39,40] However, several of the insertions found in lentiviral vectors, including internal promoters, transgenes, and regulatory elements such as the WPRE, contain large numbers of CpG dinucleotides that could be targeted by ZAP.[33–35] Unexpectedly, our data show that the CpGs in these sequences do not potently sensitize the vectors to ZAP. When ZAP is depleted in HEK293T producer cells, which is the cell type most widely used for vector production,[49] there is only an ~2-fold increase in HIV-1-based lentiviral and MLV-based gammaretroviral vector titers. Likewise, overexpression of ZAP in producer cells did not substantially inhibit vector titer. ZAP also did not restrict transduction efficiency or transgene expression in target cells. Even for a vector encoding Cas9 with the elongation factor 1a short promoter, which has many CpGs, low titer, and low transgene expression, ZAP overexpression or depletion did not substantially alter transduction efficiency.

For HIV-1, introducing CpGs into a specific region at the 5′ end of *env* potently sensitizes the virus to ZAP.[33,36,37] Importantly, this region is not within the lentiviral vector genome. However, when large numbers of CpGs were added into other regions of HIV-1, such as *gag* or *pol*, they only moderately sensitized the virus to ZAP.[33,36] Furthermore, reporter genes such as GFP or *Renilla* luciferase added to HIV-1 do not sensitize the virus to endogenous levels of ZAP, despite these genes containing large numbers of CpGs.[33,36] Therefore, it appears that CpG dinucleotides are necessary but not sufficient for ZAP to target HIV-1 RNA. This hypothesis is supported by our data showing that ZAP inhibits the titer of lentiviral vectors containing large numbers of CpGs in the internal promoter, transgene, and WPRE by only ~2-fold. It has previously been reported that ZAP depletion in THP-1 cells moderately increased the amount of lentiviral vector genomic RNA encoding Cas9 and puromycin-N-acetyltransferase

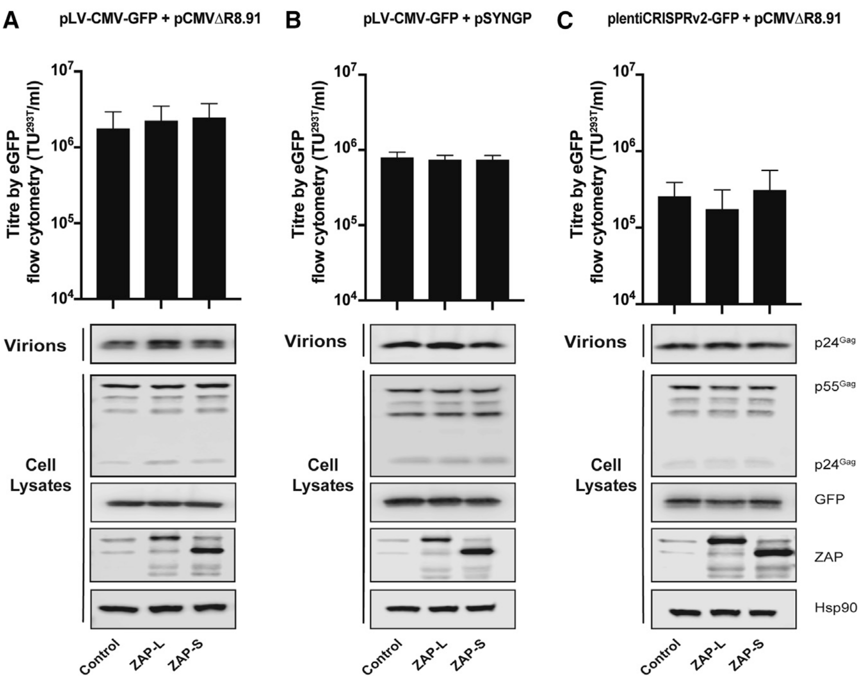

Figure 4. ZAP overexpression does not inhibit lentiviral vector titers

HEK293T cells were transfected with the indicated genome plasmid and packaging vector plus pVSV-G and pcDNA4, pHA-ZAP-L, or pHA-ZAP-S in pcDNA4. The infectious vector titer was determined by flow cytometry for GFP-positive HEK293T target cells. (A) Infectious titers of lentiviral vectors made with pLV-CMV-GFP, pCMVΔR8.91, and pVSV-G. (B) Infectious titers of lentiviral vectors made with pLV-CMV-GFP, pSYNGP, and pVSV-G. (C) infectious titers of lentiviral vectors made with plentiCRISPRv.2-GFP, pCMVΔR8.91, and pVSV-G. (A–C) Intracellular HIV-1 Gag expression from the packaging vector, GFP expression from the vector genome, HSP90, and ZAP were determined by western blots of the producer cell lysates. Virus production was determined by western blots of the producer cell supernatant. The bar charts show the average values of four (A) or three (B and C) independent experiments. Data are shown as mean ± SD.

released from these cells.[48] Whereas it is difficult to compare the different assay systems and cell lines, it should be noted that puromycin-N-acetyltransferase is a bacterial gene with a very high density of CpGs, and this may promote targeting by ZAP.[48]

Wild-type MLV has a higher CpG abundance than HIV-1 and is restricted by ZAP.[19,30,36,58] Surprisingly, ZAP did not have a larger effect on the MLV-based vector than the lentiviral vectors. ZAP has been reported to target the MLV 3′ LTR,[25] which is present in our vector genome. It should be noted that one difference between our study and previous studies showing that ZAP inhibits gammaretroviral vectors[19,24,60] is that a CMV internal promoter was present in our vector genome, whereas constructs in other studies used the U3 promoter in the 5′ LTR for transgene expression.

Because ZAP did not substantially restrict lentiviral vector production or transduction efficiency using two different packaging constructs and genome vectors, it appears that their high CpG abundance does not inherently sensitize them to ZAP. However, CpG dinucleotides in other promoters, transgenes, or regulatory elements could potentially target ZAP to the vector transcripts. Further understanding of how ZAP targets specific RNAs and inhibits gene expression could help determine if specific transgene cassettes or other inserts in retroviral vectors may sensitize them to ZAP. Recently, it has been proposed that ZAP binds with higher affinity to CpGs within a $C(n_7)G(n)CG$ context,[35] although further analysis of how this sequence promotes ZAP antiviral activity in diverse RNAs is required. In addition to binding CpGs, ZAP has also been shown to interact with UA-rich sequences in cellular and viral RNAs.[27,31,32,38] Therefore, the full range of potential ZAP binding sites in RNAs remains to be determined. It is also unknown whether there is a threshold for the number of ZAP molecules binding to an RNA for it to exert antiviral activity or whether binding sites for other RNA binding ZAP cofactors, such as TRIM25,[61,62] may also regulate its ability to inhibit gene expression. Overall, whereas ZAP strongly inhibits HIV-1 when CpGs are introduced in a specific region of the genome, increasing the CpG abundance in retroviral vectors by adding transgene expression cassettes or regulatory elements does not intrinsically sensitize the vectors to ZAP. However, it is essential to determine how ZAP specifically binds to and targets a transcript for degradation or translational inhibition so that sequences that mediate this can either be purposefully introduced or avoided in gene therapy vectors.

MATERIALS AND METHODS

Plasmids

The previously described retroviral vector genome constructs used in this study were pLV-CMV-GFP (pRRL-PPT-CMV-GFP-WPRE),[50] pMLV-CMV-GFP (pTG13077),[59] and pSEW.[56] The proviral sequence for pSEW was cloned into pGL4 (Promega), and pSEW-lowCpG was then produced by synthesizing the pSEW sequence with the non-HIV-1 regions altered to eliminate CpGs dinucleotides where possible using synonymous mutations. To generate plentiCRISPRv.2-GFP, a guide RNA targeting firefly luciferase (5′-CTT TAC CGA CGC ACA TAT CG-3′) was inserted into plentiCRISPRv.2,[54] and the puromycin-resistance gene was replaced by GFP. The HIV-1 and MLV packaging plasmids used were pCMVΔR8.91,[51] pSYNGP,[53] and pCMV-MLV Gag-Pol.[63] The following plasmids have previously been described: pGFP,[64] p-vesicular stomatitis virus G protein (pVSV-G),[65] wild-type HIV-1,[41] HIV-1$_{env86-561}$CpG,[30] p-hemagglutinin (pHA)-ZAP-L, and pHA-ZAP-S in pcDNA3.1[36] and pHA-ZAP-L and pHA-ZAP-S in pcDNA4.[24]

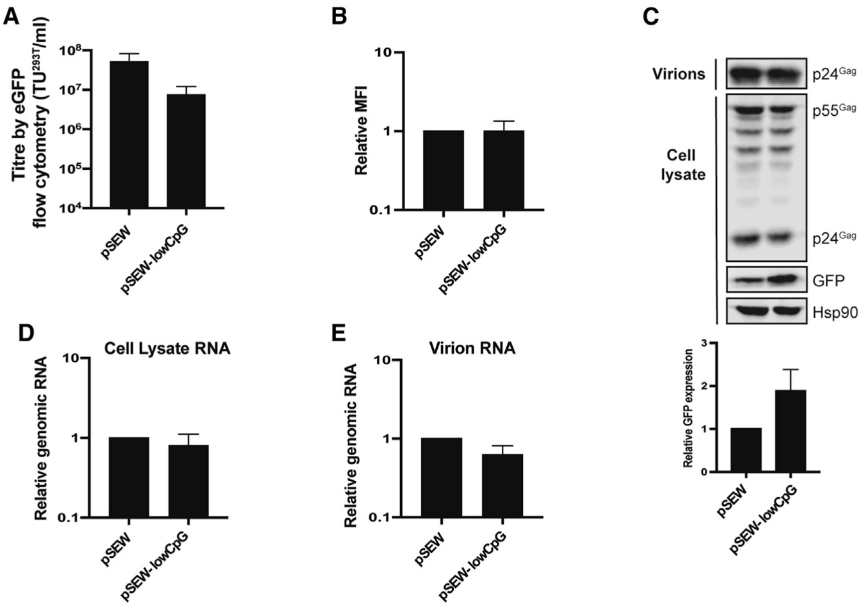

Figure 5. Removal of non-HIV-1-derived CpG dinucleotides from the lentiviral vector genome does not improve lentiviral vector titers

(A–C) HEK293T cells were transfected with pSEW or pSEW-lowCpG plus pCMVΔR8.91 and pVSV-G. (A) Jurkat T cells were transduced with the lentiviral vector supernatant. The infectious titer was determined by flow cytometry of GFP-positive cells. The bar chart shows the average values of three independent experiments. Data are shown as mean ± SD, $^{*}p < 0.05$, as determined by an unpaired t test. (B) The relative mean fluorescence intensity (MFI) of GFP-positive cells, indicating the GFP expression efficiency. The bar chart shows the average values of three independent experiments normalized to pSEW. Data are shown as mean ± SD, $^{*}p < 0.05$, as determined by an unpaired t test. (C) Expression of intracellular HIV-1 Gag from the packaging plasmid, GFP from the vector genome, and HSP90 were determined by western blotting. The band intensity of GFP was quantified for relative GFP expression. (D and E) Relative genomic RNA abundance within the producer cell lysates and virions was quantified by qRT-PCR. (B–E) The bar charts show the average values of three independent experiments normalized to pSEW.

Cells

HEK293T and TZM-bl cells[66–68] were grown in Dulbecco's modified Eagle's medium (DMEM) plus Gluta-Max (Life Technologies) supplemented with 10% fetal bovine serum (FBS) and 1% penicillin-streptomycin. Jurkat cells were grown in RPMI (Life Technologies) supplemented with 10% FBS and 1% penicillin-streptomycin. All cells were maintained in a humidified atmosphere with 5% CO_2 at 37°C.

Control and ZAP knockout HEK293T cell lines were produced by CRISPR-Cas9-mediated genome editing. Firefly luciferase or ZAP targeting guide sequences were inserted into plentiCRISPRv.2.[54] The CRISPR guide sequences are firefly luciferase-G1 (control): 5′-CTT TAC CGA CGC ACA TAT CG-3′; ZAP-ex4: 5′-TCT GGT AGA AGT TAT ATC TG-3′; and ZAP-ex6: 5′-ACT TCC ATC TGC CTT 622 ACC GG-3′. Viral stocks were produced in HEK293T cells by transfection with pCMVΔR8.91, plentiCRISPRv.2, and pVSV-G at a ratio of 1:1:0.5 using 1 mg/mL polyethyleneimine (PEI) at a DNA:PEI ratio of 1:3. The supernatant was harvested and filtered 48 h post-transfection through a 0.45-μm filter (Millipore). HEK293T cells were transduced with the plentiCRISPRv.2 virus and cultured for 5–7 days in 1 μg/mL puromycin. ZAP depletion was validated by western blotting.

Retroviral vector production

HEK293T cells were seeded 24 h prior to transfection at a density of 10^6 cells per well in a six-well plate. Plasmids for transfection were prepared in Opti-MEM. Each well was co-transfected with 1 μg vector genome, 1 μg packaging plasmid, and 0.5 μg pVSV-G using PEI at a DNA/PEI ratio of 1:3. For ZAP overexpression experiments, 0.5 μg of pZAP-L, pZAP-S, or pCDNA4 vector was also transfected. Media were changed 6 h post-transfection, and the supernatant containing viral vector particles was filtered through a 0.45-μm filter 48 h post-transfection.

Determination of retroviral vector titer

HEK293T or Jurkat cells were plated in 96-well plates as target cells for transduction. A serial dilution of supernatant containing viral vectors was prepared and added to the wells. Cells were harvested 48 h post-transduction, fixed in 2% paraformaldehyde, and re-suspended in 1× phosphate-buffered saline (PBS). Samples were run on a flow cytometer to detect GFP-positive cells, which was used to calculate the titer (transducing units per milliliter).

Quantification of vector titer by ddPCR was carried out by transducing HEK293T cells in suspension with a serial dilution of supernatant containing lentiviral vectors. 3 days after transduction, the cells were lysed, and DNA was extracted using the DNeasy Blood and Tissue Kit (QIAGEN). ddPCR was performed using the following primer probe set to quantify the number of vector genomes: forward 5′-TCTCGACGCAGGACTCG-3′, reverse 5′-CGCTCTCGCACC CATCTC-3′, and probe 5′-FAM-CTCCTTCTAGCCTCCGCTAG-BHQ1-3′.

Primary cell isolation, activation, and transduction

Human primary CD4$^+$ T cells were obtained from peripheral blood mononuclear cells (PBMCs) from healthy volunteer donors through the Infectious Diseases BioBank at King's College London (ethics reference MM2-220518) under overall permission from the Southampton and South West Hampshire Research Ethics Committee (REC; B) (REC reference 19/SC/0232). PBMCs were isolated using density gradient centrifugation in SepMate tubes (STEMCELL Technologies) with Lymphoprep density gradient medium (STEMCELL Technologies). Total CD4$^+$ T cells were isolated using a Human CD4$^+$ T Cell Isolation Kit (Miltenyi Biotec). CD4$^+$ T cells were cultured in RPMI-1640 medium with GlutaMAX and HEPES supplemented with 10% heat-inactivated autologous human serum and 1%

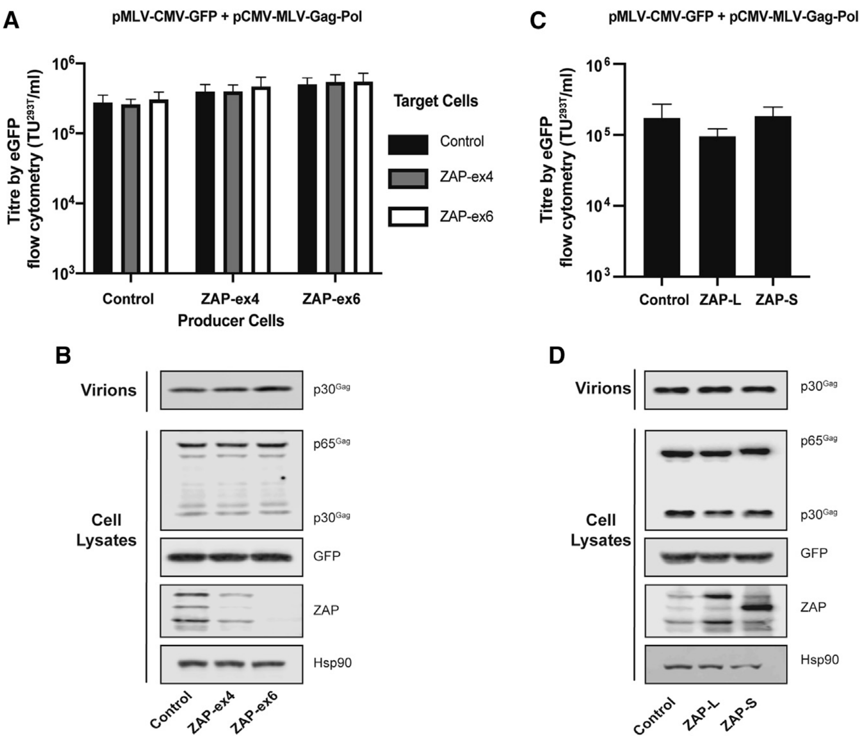

Figure 6. ZAP does not substantially inhibit a gammaretroviral vector

(A) HEK293T CRISPR control, ZAP-ex4 ,or ZAP-ex6 producer cells were transfected with pMLV-CMV-GFP, pCMV-MLV Gag-Pol, and pVSV-G. Infectious titers were determined in transduced target cells (HEK293T CRISPR control, ZAP-ex4, or ZAP-ex6 cells) by flow cytometry for GFP-positive cells. (B and D) Intracellular MLV Gag expression from the packaging plasmid, GFP expression from the vector genome, and HSP90 and ZAP expression were determined by western blots of the producer cell lysates. Virus production was determined by western blots of the producer cell supernatant. (C) HEK293T cells were transfected with the pMLV-CMV-GFP genome and pCMV-MLV Gag-Pol packaging vector plus pVSV-G and pcDNA4, pZAP-L, or pZAP-S. Infectious titers of the vectors were determined by flow cytometry for GFP-positive HEK293T target cells. (A and C) The bar charts show the average values of three independent experiments. Data are shown as mean ± SD.

penicillin-streptomycin (Life Technologies). Cells were then activated using Dynabeads Human T-Activator CD3/CD28 (Life Technologies) and recombinant human interleukin-2 (30 U/mL; Roche) for 48 h prior to infection.

Activated $CD4^+$ T cells were transduced with lentiviral vectors (1−10 ng of $p24^{Gag}$ of vector per 50,000 cells). 48 h post-transduction, the cells were fixed in 4% paraformaldehyde in DPBS before assessing transduction efficiency by flow cytometry measuring intracellular GFP expression.

Quantitative RT-PCR

Cells were washed with 1× PBS, and the RNA was extracted using the RNeasy mini kit (QIAGEN) following the manufacturer's instructions. To extract virion RNA, the supernatant was spun through a 20% sucrose cushion in 1× PBS to pellet the vector particles. Virion RNA was extracted using the QIAamp Viral Mini Kit (QIAGEN). 1 μg of cellular RNA and 20 μL of virion RNA were reverse transcribed using the High Capacity cDNA Reverse-Transcription kit (Applied Biosystems). Quantitative PCR was performed using the Taqman Universal PCR Mix and the QuantiStudio 5 System (Thermo Fisher Scientific). Absolute quantification for genomic RNA abundance was determined using a standard curve of the lentiviral vector DNA plasmid for both the cell lysate and virion RNA samples. The genomic RNA primers were 5′-TCTCGACGCAGGACTCG-3′/5′-TACTGACGCTCTCGC ACC-3′ (forward/reverse), and the probe was 5′-FAM-ATC TCTCTCCTTCTAGCCTC-TAMRA-3′.

HIV-1 infection assays

HEK293T cells in six-well plates were transfected according to the manufacturer's instructions using PEI (1 mg/mL) (Sigma-Aldrich) at the ratio of 4 μL PEI to1 μg DNA. 0.5 μg pHIV-1 and 0.5 μg pGFP were transfected for a total of 1 μg DNA. The transfection medium was replaced with fresh medium after 6 h, and this medium was recovered ∼48 h post-transfection. To generate cell-free virus stocks, the media were filtered through 0.45 μM filters (Millipore). Infectious virus was quantified using TZM-bl cells. These cells were seeded in 24-well plates and incubated overnight with virus stocks. 48 h post-infection, the cells were lysed, and infectivity was measured using the Galacto-Star β-galactosidase system according to the manufacturer's instructions (Applied Biosystems). A PerkinElmer luminometer was used to quantify β-galactosidase activity as relative light units per second.

Western blotting

48 h post-transfection, cells were lysed in radioimmunoprecipitation (RIPA) buffer (10 mM Tris-HCl, pH 7.5, 150 mM NaCl, 1 mM EDTA, 0.1% sodium dodecyl sulfate (SDS), 1% Triton X-100, 1% sodium deoxycholate). The supernatant was filtered through a 0.45-μm filter. Virions were pelleted through a 20% sucrose cushion in PBS solution for 2 h at 20,000 × *g*. The pellet was resuspended in 2× loading buffer (60 mM Tris-HCl, pH 6.8, 10% β-mercaptoethanol, 10% glycerol, 2% SDS, 0.1% bromophenol blue). Cell lysates and virions were resolved by SDS-polyacrylamide gel electrophoresis and transferred to a nitrocellulose membrane. Protein bands were detected using the LI-COR infrared imaging system (LI-COR UK).

The antibodies used in this study were 1:50 HIV-1 anti-$p24^{Gag}$ (183-H12-5C),[69] 1:1,000 anti-heat shock protein (HSP)90 (Santa Cruz Biotechnology; sc7947), 1:5,000 anti-ZAP (Abcam; ab154680),

1:1,000 anti-GFP (Sigma-Aldrich; 11814460001), 1:10,000 anti-MLV p30^{Gag} (ATCC; CRL-1912)[70], 1:10,000 Dylight 800-conjugated anti-mouse/rabbit secondary antibodies (Cell Signaling Technology), and IRDye 800CW goat anti-rat immunoglobulin G (IgG; LI-COR Biosciences).

Enzyme-linked immunosorbent assay

Gag (p24^{Gag}) was quantified using the AlphaLISA HIV-p24 (high sensitivity) Kit (PerkinElmer), according to the manufacturer's instructions. Luminescence levels were measured with the EnVision Multimode plate reader (PerkinElmer), and p24^{Gag} levels interpolated from the linear portion of the standard curve.

Sequence analysis

The CG dinucleotide frequencies for the viral vector genomes (from the 5′R to the 3′R) were determined using the "analyze base composition" tool in MacVector (MacVector).

SUPPLEMENTAL INFORMATION

Supplemental information can be found online at https://doi.org/10.1016/j.omtm.2021.08.008.

ACKNOWLEDGMENTS

We thank the members of the Swanson and Neil labs as well as Luis Apolonia for helpful discussions. We also thank Luis Apolonia, Caroline Goujon, Harmit Malik, and Jonathan Stoye for generously providing reagents. The following reagents were obtained through the NIH AIDS Research and Reference Reagent Program, Division of AIDS, NIAID, NIH: TZM-bl from Dr. John C. Kappes, Dr. Xiaoyun Wu, and Tranzyme and HIV-1 p24 hybridoma (183-H12-5C; cat. #1513) from Dr. Bruce Chesebro. These studies were funded by MRC Discovery Award MC/PC/15068 and a Wellcome Trust Senior Research Fellowship (WT098049AIA) to S.J.D.N., Wellcome Trust (106223/Z/14/Z) to M.H.M., Medical Research Council grant MR/M019756/1 to C.M.S., and Medical Research Council grant MR/S000844/1 to S.J.D.N. and C.M.S. MR/S000844/1 is part of the EDCTP2 Program supported by the European Union. H.S. is supported by the BBSRC Industrial CASE Partnerships (ICP) training grant (BB/P504609/1) with GSK. M.F. and A.W.S. were supported by the MRC-KCL Doctoral Training Partnership in Biomedical Sciences (MR/R50225X/1 and MR/N013700/1). D.K. is funded by Deutsche Forschungsgemeinschaft (DFG [German Research Foundation]) project number: KM 5/1-1. This work was also supported via a National Institute for Health Research Biomedical Research Centre Award to Guy's and St Thomas' NHS Foundation Trust in partnership with King's College London.

AUTHOR CONTRIBUTIONS

Conceptualization, C.M.S.; investigation, H.S., L.H., M.F., D.K., A.W.S., and S.A.; visualization, H.S., L.H., M.F., and D.K.; writing – original draft, C.M.S.; writing – review & editing, H.S., L.H., M.F., D.K., A.W.S., H.W., S.J.D.N., M.H.M., C.A.V., and C.M.S.; funding acquisition, C.A.V. and C.M.S.; resources, H.P., H.W., and S.J.D.N.; supervision, M.H.M., C.A.V., and C.M.S.

DECLARATION OF INTERESTS

The authors declare no competing interests.

REFERENCES

1. Dunbar, C.E., High, K.A., Joung, J.K., Kohn, D.B., Ozawa, K., and Sadelain, M. (2018). Gene therapy comes of age. Science *359*, eaan4672.
2. Hoggatt, J. (2016). Gene Therapy for "Bubble Boy" Disease. Cell *166*, 263.
3. Sadelain, M. (2017). CD19 CAR T Cells. Cell *171*, 1471.
4. Chemudupati, M., Kenney, A.D., Bonifati, S., Zani, A., McMichael, T.M., Wu, L., and Yount, J.S. (2019). From APOBEC to ZAP: Diverse mechanisms used by cellular restriction factors to inhibit virus infections. Biochim. Biophys. Acta Mol. Cell Res. *1866*, 382–394.
5. Borsotti, C., Borroni, E., and Follenzi, A. (2016). Lentiviral vector interactions with the host cell. Curr. Opin. Virol. *21*, 102–108.
6. Sheehy, A.M., Gaddis, N.C., Choi, J.D., and Malim, M.H. (2002). Isolation of a human gene that inhibits HIV-1 infection and is suppressed by the viral Vif protein. Nature *418*, 646–650.
7. Neil, S.J., Zang, T., and Bieniasz, P.D. (2008). Tetherin inhibits retrovirus release and is antagonized by HIV-1 Vpu. Nature *451*, 425–430.
8. Wiegand, H.L., Doehle, B.P., Bogerd, H.P., and Cullen, B.R. (2004). A second human antiretroviral factor, APOBEC3F, is suppressed by the HIV-1 and HIV-2 Vif proteins. EMBO J. *23*, 2451–2458.
9. Goujon, C., Moncorgé, O., Bauby, H., Doyle, T., Ward, C.C., Schaller, T., Hué, S., Barclay, W.S., Schulz, R., and Malim, M.H. (2013). Human MX2 is an interferon-induced post-entry inhibitor of HIV-1 infection. Nature *502*, 559–562.
10. Kane, M., Yadav, S.S., Bitzegeio, J., Kutluay, S.B., Zang, T., Wilson, S.J., Schoggins, J.W., Rice, C.M., Yamashita, M., Hatziioannou, T., and Bieniasz, P.D. (2013). MX2 is an interferon-induced inhibitor of HIV-1 infection. Nature *502*, 563–566.
11. Liu, Z., Pan, Q., Ding, S., Qian, J., Xu, F., Zhou, J., Cen, S., Guo, F., and Liang, C. (2013). The interferon-inducible MxB protein inhibits HIV-1 infection. Cell Host Microbe *14*, 398–410.
12. Hrecka, K., Hao, C., Gierszewska, M., Swanson, S.K., Kesik-Brodacka, M., Srivastava, S., Florens, L., Washburn, M.P., and Skowronski, J. (2011). Vpx relieves inhibition of HIV-1 infection of macrophages mediated by the SAMHD1 protein. Nature *474*, 658–661.
13. Laguette, N., Sobhian, B., Casartelli, N., Ringeard, M., Chable-Bessia, C., Ségéral, E., Yatim, A., Emiliani, S., Schwartz, O., and Benkirane, M. (2011). SAMHD1 is the dendritic- and myeloid-cell-specific HIV-1 restriction factor counteracted by Vpx. Nature *474*, 654–657.
14. Stremlau, M., Owens, C.M., Perron, M.J., Kiessling, M., Autissier, P., and Sodroski, J. (2004). The cytoplasmic body component TRIM5alpha restricts HIV-1 infection in Old World monkeys. Nature *427*, 848–853.
15. Schoggins, J.W., Wilson, S.J., Panis, M., Murphy, M.Y., Jones, C.T., Bieniasz, P., and Rice, C.M. (2011). A diverse range of gene products are effectors of the type I interferon antiviral response. Nature *472*, 481–485.
16. Jimenez-Guardeño, J.M., Apolonia, L., Betancor, G., and Malim, M.H. (2019). Immunoproteasome activation enables human TRIM5α restriction of HIV-1. Nat. Microbiol. *4*, 933–940.
17. Petrillo, C., Thorne, L.G., Unali, G., Schiroli, G., Giordano, A.M.S., Piras, F., Cuccovillo, I., Petit, S.J., Ahsan, F., Noursadeghi, M., et al. (2018). Cyclosporine H Overcomes Innate Immune Restrictions to Improve Lentiviral Transduction and Gene Editing In Human Hematopoietic Stem Cells. Cell Stem Cell *23*, 820–832.e9.
18. Ficarelli, M., Neil, S.J.D., and Swanson, C.M. (2021). Targeted Restriction of Viral Gene Expression and Replication by the ZAP Antiviral System. Annu. Rev. Virol. Published online June 15, 2021. https://doi.org/10.1146/annurev-virology-091919-104213.

19. Gao, G., Guo, X., and Goff, S.P. (2002). Inhibition of retroviral RNA production by ZAP, a CCCH-type zinc finger protein. Science *297*, 1703–1706.

20. Bick, M.J., Carroll, J.W., Gao, G., Goff, S.P., Rice, C.M., and MacDonald, M.R. (2003). Expression of the zinc-finger antiviral protein inhibits alphavirus replication. J. Virol. *77*, 11555–11562.

21. Guo, X., Ma, J., Sun, J., and Gao, G. (2007). The zinc-finger antiviral protein recruits the RNA processing exosome to degrade the target mRNA. Proc. Natl. Acad. Sci. USA *104*, 151–156.

22. Zhu, Y., Wang, X., Goff, S.P., and Gao, G. (2012). Translational repression precedes and is required for ZAP-mediated mRNA decay. EMBO J. *31*, 4236–4246.

23. Li, M.M.H., Aguilar, E.G., Michailidis, E., Pabon, J., Park, P., Wu, X., de Jong, Y.P., Schneider, W.M., Molina, H., Rice, C.M., and MacDonald, M.R. (2019). Characterization of Novel Splice Variants of Zinc Finger Antiviral Protein (ZAP). J. Virol. *93*, e00715–e00719.

24. Kerns, J.A., Emerman, M., and Malik, H.S. (2008). Positive selection and increased antiviral activity associated with the PARP-containing isoform of human zinc-finger antiviral protein. PLoS Genet. *4*, e21.

25. Guo, X., Carroll, J.W., Macdonald, M.R., Goff, S.P., and Gao, G. (2004). The zinc finger antiviral protein directly binds to specific viral mRNAs through the CCCH zinc finger motifs. J. Virol. *78*, 12781–12787.

26. Charron, G., Li, M.M., MacDonald, M.R., and Hang, H.C. (2013). Prenylome profiling reveals S-farnesylation is crucial for membrane targeting and antiviral activity of ZAP long-isoform. Proc. Natl. Acad. Sci. USA *110*, 11085–11090.

27. Schwerk, J., Soveg, F.W., Ryan, A.P., Thomas, K.R., Hatfield, L.D., Ozarkar, S., Forero, A., Kell, A.M., Roby, J.A., So, L., et al. (2019). RNA-binding protein isoforms ZAP-S and ZAP-L have distinct antiviral and immune resolution functions. Nat. Immunol. *20*, 1610–1620.

28. Li, M.M., Lau, Z., Cheung, P., Aguilar, E.G., Schneider, W.M., Bozzacco, L., Molina, H., Buehler, E., Takaoka, A., Rice, C.M., et al. (2017). TRIM25 Enhances the Antiviral Action of Zinc-Finger Antiviral Protein (ZAP). PLoS Pathog. *13*, e1006145.

29. Zheng, X., Wang, X., Tu, F., Wang, Q., Fan, Z., and Gao, G. (2017). TRIM25 Is Required for the Antiviral Activity of Zinc Finger Antiviral Protein. J. Virol. *91*, e00088-17.

30. Ficarelli, M., Wilson, H., Pedro Galão, R., Mazzon, M., Antzin-Anduetza, I., Marsh, M., Neil, S.J., and Swanson, C.M. (2019). KHNYN is essential for the zinc finger antiviral protein (ZAP) to restrict HIV-1 containing clustered CpG dinucleotides. eLife *8*, e46767.

31. Odon, V., Fros, J.J., Goonawardane, N., Dietrich, I., Ibrahim, A., Alshaikhahmed, K., Nguyen, D., and Simmonds, P. (2019). The role of ZAP and OAS3/RNAseL pathways in the attenuation of an RNA virus with elevated frequencies of CpG and UpA dinucleotides. Nucleic Acids Res. *47*, 8061–8083.

32. Goonawardane, N., Nguyen, D., and Simmonds, P. (2021). Association of Zinc Finger Antiviral Protein Binding to Viral Genomic RNA with Attenuation of Replication of Echovirus 7. MSphere *6*, e01138-20.

33. Takata, M.A., Gonçalves-Carneiro, D., Zang, T.M., Soll, S.J., York, A., Blanco-Melo, D., and Bieniasz, P.D. (2017). CG dinucleotide suppression enables antiviral defence targeting non-self RNA. Nature *550*, 124–127.

34. Meagher, J.L., Takata, M., Gonçalves-Carneiro, D., Keane, S.C., Rebendenne, A., Ong, H., Orr, V.K., MacDonald, M.R., Stuckey, J.A., Bieniasz, P.D., and Smith, J.L. (2019). Structure of the zinc-finger antiviral protein in complex with RNA reveals a mechanism for selective targeting of CG-rich viral sequences. Proc. Natl. Acad. Sci. USA *116*, 24303–24309.

35. Luo, X., Wang, X., Gao, Y., Zhu, J., Liu, S., Gao, G., and Gao, P. (2020). Molecular Mechanism of RNA Recognition by Zinc-Finger Antiviral Protein. Cell Rep. *30*, 46–52.e4.

36. Ficarelli, M., Antzin-Anduetza, I., Hugh-White, R., Firth, A.E., Sertkaya, H., Wilson, H., Neil, S.J.D., Schulz, R., and Swanson, C.M. (2020). CpG Dinucleotides Inhibit HIV-1 Replication through Zinc Finger Antiviral Protein (ZAP)-Dependent and -Independent Mechanisms. J. Virol. *94*, e01337-19.

37. Kmiec, D., Nchioua, R., Sherrill-Mix, S., Stürzel, C.M., Heusinger, E., Braun, E., Gondim, M.V.P., Hotter, D., Sparrer, K.M.J., Hahn, B.H., et al. (2020). CpG Frequency in the 5′ Third of the *env* Gene Determines Sensitivity of Primary HIV-1 Strains to the Zinc-Finger Antiviral Protein. MBio *11*, e02903–e02919.

38. Fros, J.J., Visser, I., Tang, B., Yan, K., Nakayama, E., Visser, T.M., Koenraadt, C.J.M., van Oers, M.M., Pijlman, G.P., Suhrbier, A., and Simmonds, P. (2021). The dinucleotide composition of the Zika virus genome is shaped by conflicting evolutionary pressures in mammalian hosts and mosquito vectors. PLoS Biol. *19*, e3001201.

39. Kypr, J., Mrázek, J., and Reich, J. (1989). Nucleotide composition bias and CpG dinucleotide content in the genomes of HIV and HTLV 1/2. Biochim. Biophys. Acta *1009*, 280–282.

40. Shpaer, E.G., and Mullins, J.I. (1990). Selection against CpG dinucleotides in lentiviral genes: a possible role of methylation in regulation of viral expression. Nucleic Acids Res. *18*, 5793–5797.

41. Antzin-Anduetza, I., Mahiet, C., Granger, L.A., Odendall, C., and Swanson, C.M. (2017). Increasing the CpG dinucleotide abundance in the HIV-1 genomic RNA inhibits viral replication. Retrovirology *14*, 49.

42. Karlin, S., Doerfler, W., and Cardon, L.R. (1994). Why is CpG suppressed in the genomes of virtually all small eukaryotic viruses but not in those of large eukaryotic viruses? J. Virol. *68*, 2889–2897.

43. Alinejad-Rokny, H., Anwar, F., Waters, S.A., Davenport, M.P., and Ebrahimi, D. (2016). Source of CpG Depletion in the HIV-1 Genome. Mol. Biol. Evol. *33*, 3205–3212.

44. Naldini, L., Blömer, U., Gage, F.H., Trono, D., and Verma, I.M. (1996). Efficient transfer, integration, and sustained long-term expression of the transgene in adult rat brains injected with a lentiviral vector. Proc. Natl. Acad. Sci. USA *93*, 11382–11388.

45. Zufferey, R., Donello, J.E., Trono, D., and Hope, T.J. (1999). Woodchuck hepatitis virus posttranscriptional regulatory element enhances expression of transgenes delivered by retroviral vectors. J. Virol. *73*, 2886–2892.

46. Timms, R.T., Tchasovnikarova, I.A., and Lehner, P.J. (2016). Position-effect variegation revisited: HUSHing up heterochromatin in human cells. BioEssays *38*, 333–343.

47. Emery, D.W. (2011). The use of chromatin insulators to improve the expression and safety of integrating gene transfer vectors. Hum. Gene Ther. *22*, 761–774.

48. OhAinle, M., Helms, L., Vermeire, J., Roesch, F., Humes, D., Basom, R., Delrow, J.J., Overbaugh, J., and Emerman, M. (2018). A virus-packageable CRISPR screen identifies host factors mediating interferon inhibition of HIV. eLife *7*, e39823.

49. Ferreira, M.V., Cabral, E.T., and Coroadinha, A.S. (2021). Progress and Perspectives in the Development of Lentiviral Vector Producer Cells. Biotechnol. J. *16*, e2000017.

50. Jarrosson-Wuilleme, L., Goujon, C., Bernaud, J., Rigal, D., Darlix, J.L., and Cimarelli, A. (2006). Transduction of nondividing human macrophages with gammaretrovirus-derived vectors. J. Virol. *80*, 1152–1159.

51. Zufferey, R., Nagy, D., Mandel, R.J., Naldini, L., and Trono, D. (1997). Multiply attenuated lentiviral vector achieves efficient gene delivery in vivo. Nat. Biotechnol. *15*, 871–875.

52. Wagner, R., Graf, M., Bieler, K., Wolf, H., Grunwald, T., Foley, P., and Uberla, K. (2000). Rev-independent expression of synthetic gag-pol genes of human immunodeficiency virus type 1 and simian immunodeficiency virus: implications for the safety of lentiviral vectors. Hum. Gene Ther. *11*, 2403–2413.

53. Kotsopoulou, E., Kim, V.N., Kingsman, A.J., Kingsman, S.M., and Mitrophanous, K.A. (2000). A Rev-independent human immunodeficiency virus type 1 (HIV-1)-based vector that exploits a codon-optimized HIV-1 gag-pol gene. J. Virol. *74*, 4839–4852.

54. Sanjana, N.E., Shalem, O., and Zhang, F. (2014). Improved vectors and genome-wide libraries for CRISPR screening. Nat. Methods *11*, 783–784.

55. Zhu, Y., Chen, G., Lv, F., Wang, X., Ji, X., Xu, Y., Sun, J., Wu, L., Zheng, Y.T., and Gao, G. (2011). Zinc-finger antiviral protein inhibits HIV-1 infection by selectively targeting multiply spliced viral mRNAs for degradation. Proc. Natl. Acad. Sci. USA *108*, 15834–15839.

56. Demaison, C., Parsley, K., Brouns, G., Scherr, M., Battmer, K., Kinnon, C., Grez, M., and Thrasher, A.J. (2002). High-level transduction and gene expression in hematopoietic repopulating cells using a human immunodeficiency [correction of imunodeficiency] virus type 1-based lentiviral vector containing an internal spleen focus forming virus promoter. Hum. Gene Ther. *13*, 803–813.

57. Bauer, A.P., Leikam, D., Krinner, S., Notka, F., Ludwig, C., Längst, G., and Wagner, R. (2010). The impact of intragenic CpG content on gene expression. Nucleic Acids Res. *38*, 3891–3908.

58. Lee, H., Komano, J., Saitoh, Y., Yamaoka, S., Kozaki, T., Misawa, T., Takahama, M., Satoh, T., Takeuchi, O., Yamamoto, N., et al. (2013). Zinc-finger antiviral protein mediates retinoic acid inducible gene I-like receptor-independent antiviral response to murine leukemia virus. Proc. Natl. Acad. Sci. USA *110*, 12379–12384.

59. Nègre, D., Mangeot, P.E., Duisit, G., Blanchard, S., Vidalain, P.O., Leissner, P., Winter, A.J., Rabourdin-Combe, C., Mehtali, M., Moullier, P., et al. (2000). Characterization of novel safe lentiviral vectors derived from simian immunodeficiency virus (SIVmac251) that efficiently transduce mature human dendritic cells. Gene Ther. *7*, 1613–1623.

60. Wang, X., Tu, F., Zhu, Y., and Gao, G. (2012). Zinc-finger antiviral protein inhibits XMRV infection. PLoS ONE *7*, e39159.

61. Choudhury, N.R., Heikel, G., Trubitsyna, M., Kubik, P., Nowak, J.S., Webb, S., Granneman, S., Spanos, C., Rappsilber, J., Castello, A., and Michlewski, G. (2017). RNA-binding activity of TRIM25 is mediated by its PRY/SPRY domain and is required for ubiquitination. BMC Biol. *15*, 105.

62. Sanchez, J.G., Sparrer, K.M.J., Chiang, C., Reis, R.A., Chiang, J.J., Zurenski, M.A., Wan, Y., Gack, M.U., and Pornillos, O. (2018). TRIM25 Binds RNA to Modulate Cellular Anti-viral Defense. J. Mol. Biol. *430*, 5280–5293.

63. Soneoka, Y., Cannon, P.M., Ramsdale, E.E., Griffiths, J.C., Romano, G., Kingsman, S.M., and Kingsman, A.J. (1995). A transient three-plasmid expression system for the production of high titer retroviral vectors. Nucleic Acids Res. *23*, 628–633.

64. Swanson, C.M., Sherer, N.M., and Malim, M.H. (2010). SRp40 and SRp55 promote the translation of unspliced human immunodeficiency virus type 1 RNA. J. Virol. *84*, 6748–6759.

65. Fouchier, R.A., Meyer, B.E., Simon, J.H., Fischer, U., and Malim, M.H. (1997). HIV-1 infection of non-dividing cells: evidence that the amino-terminal basic region of the viral matrix protein is important for Gag processing but not for post-entry nuclear import. EMBO J. *16*, 4531–4539.

66. Derdeyn, C.A., Decker, J.M., Sfakianos, J.N., Wu, X., O'Brien, W.A., Ratner, L., Kappes, J.C., Shaw, G.M., and Hunter, E. (2000). Sensitivity of human immunodeficiency virus type 1 to the fusion inhibitor T-20 is modulated by coreceptor specificity defined by the V3 loop of gp120. J. Virol. *74*, 8358–8367.

67. Wei, X., Decker, J.M., Liu, H., Zhang, Z., Arani, R.B., Kilby, J.M., Saag, M.S., Wu, X., Shaw, G.M., and Kappes, J.C. (2002). Emergence of resistant human immunodeficiency virus type 1 in patients receiving fusion inhibitor (T-20) monotherapy. Antimicrob. Agents Chemother. *46*, 1896–1905.

68. Platt, E.J., Wehrly, K., Kuhmann, S.E., Chesebro, B., and Kabat, D. (1998). Effects of CCR5 and CD4 cell surface concentrations on infections by macrophagetropic isolates of human immunodeficiency virus type 1. J. Virol. *72*, 2855–2864.

69. Chesebro, B., Wehrly, K., Nishio, J., and Perryman, S. (1992). Macrophage-tropic human immunodeficiency virus isolates from different patients exhibit unusual V3 envelope sequence homogeneity in comparison with T-cell-tropic isolates: definition of critical amino acids involved in cell tropism. J. Virol. *66*, 6547–6554.

70. Chesebro, B., Britt, W., Evans, L., Wehrly, K., Nishio, J., and Cloyd, M. (1983). Characterization of monoclonal antibodies reactive with murine leukemia viruses: use in analysis of strains of friend MCF and Friend ecotropic murine leukemia virus. Virology *127*, 134–148.

OMTM, Volume *23*

Supplemental information

Minimal impact of ZAP on lentiviral vector production and transduction efficiency

Helin Sertkaya, Laura Hidalgo, Mattia Ficarelli, Dorota Kmiec, Adrian W. Signell, Sadfer Ali, Hannah Parker, Harry Wilson, Stuart J.D. Neil, Michael H. Malim, Conrad A. Vink, and Chad M. Swanson

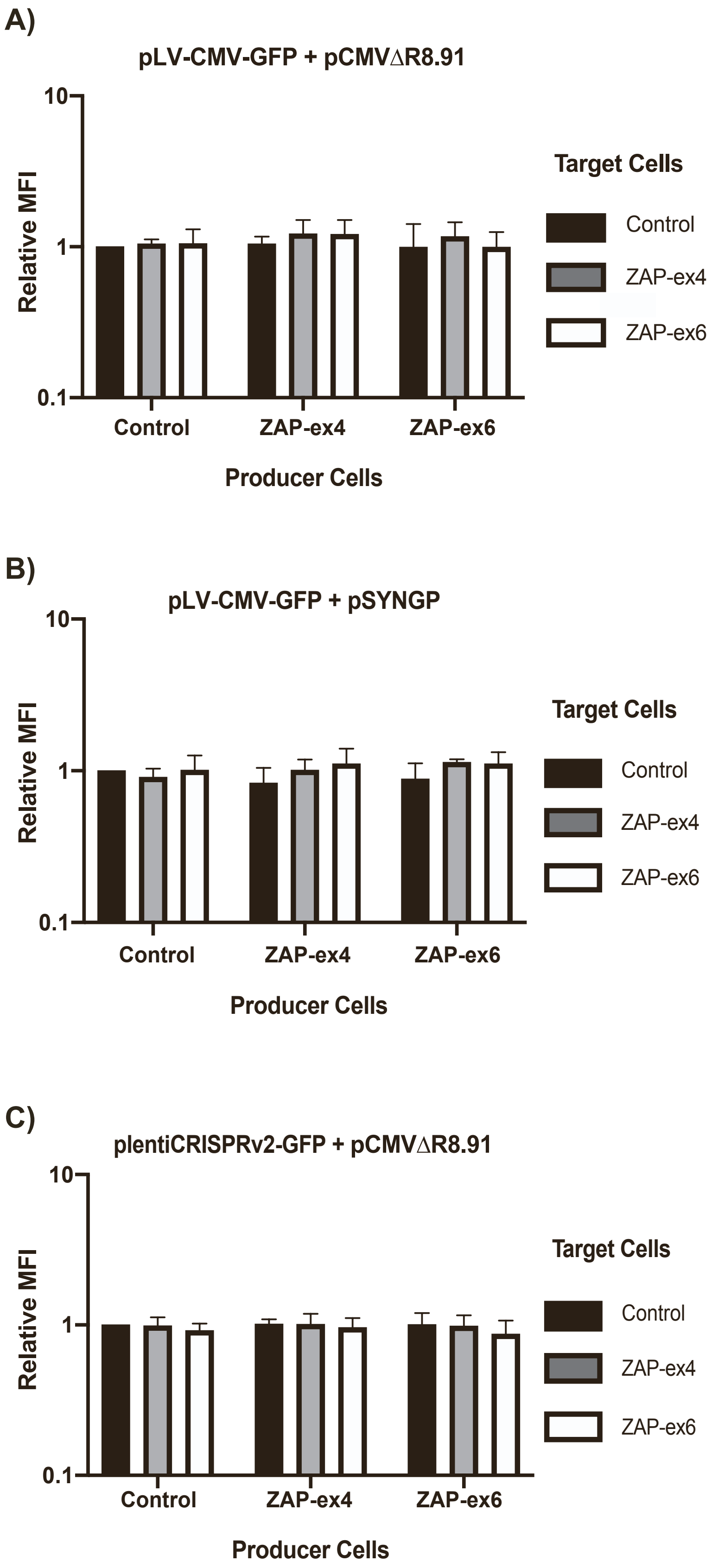

Figure S1. ZAP depletion does not affect transgene expression in a lentiviral vector.

Media from HEK293T producer cells (CRISPR control, ZAP-ex4 or ZAP-ex6) transfected with the indicated genome plasmid, packaging plasmid and pVSV-G was used to transduce target cells (HEK293T CRISPR control, ZAP-ex4 or ZAP-ex6 cells). MFI was determined by flow cytometry for GFP-positive target cells. The bar charts show the average values of three independent experiments. Data are shown as mean ± SD.

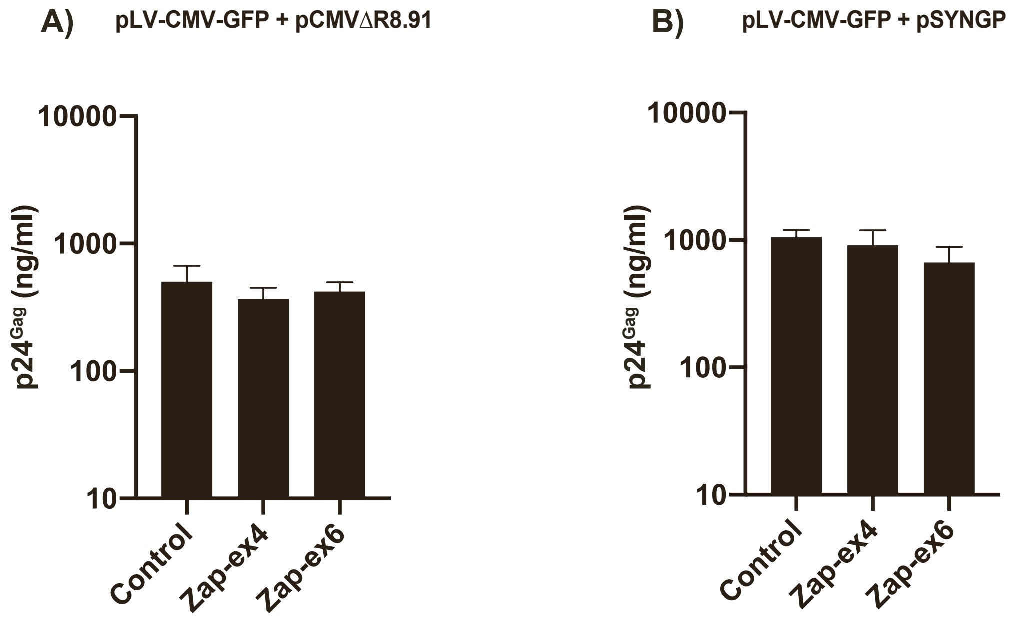

Figure S2. ZAP depletion does not affect $p24^{Gag}$ content in lentiviral vectors.

Media from HEK293T producer cells (CRISPR control, ZAP-ex4 or ZAP-ex6) transfected with the indicated genome and packaging plasmid and pVSV-G was used to quantify HIV-1 Capsid ($p24^{Gag}$) levels. The bar charts show the average values of three independent experiments. Data shown as mean ± SD.

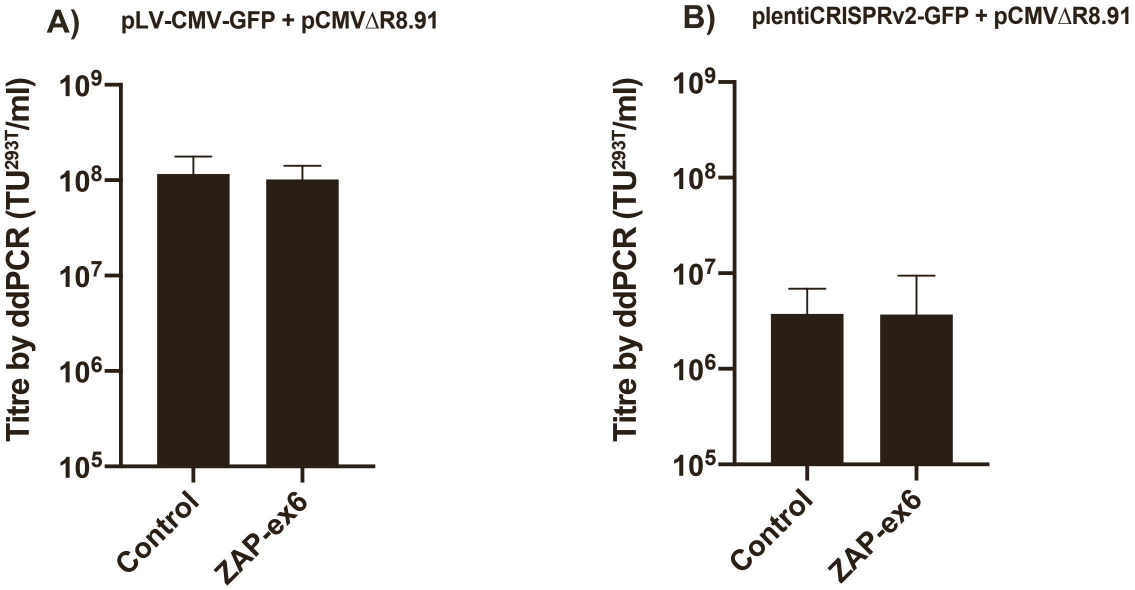

Figure S3. Endogenous ZAP in HEK293T cells does not restrict lentiviral vector titres measured by ddPCR

Media from HEK293T producer cells (CRISPR control or ZAP-ex6) transfected with the indicated genome plasmid, pCMVΔR8.91 and pVSV-G was used to transduce HEK293T cells. Infectious titres were determined in transduced cells by digital droplet PCR (ddPCR). Data shown as mean ± SD.

A)

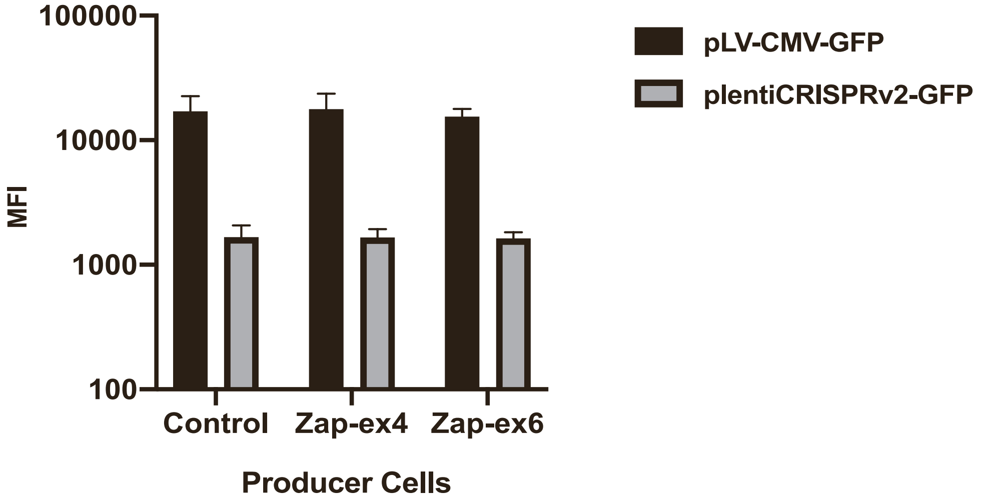

B)

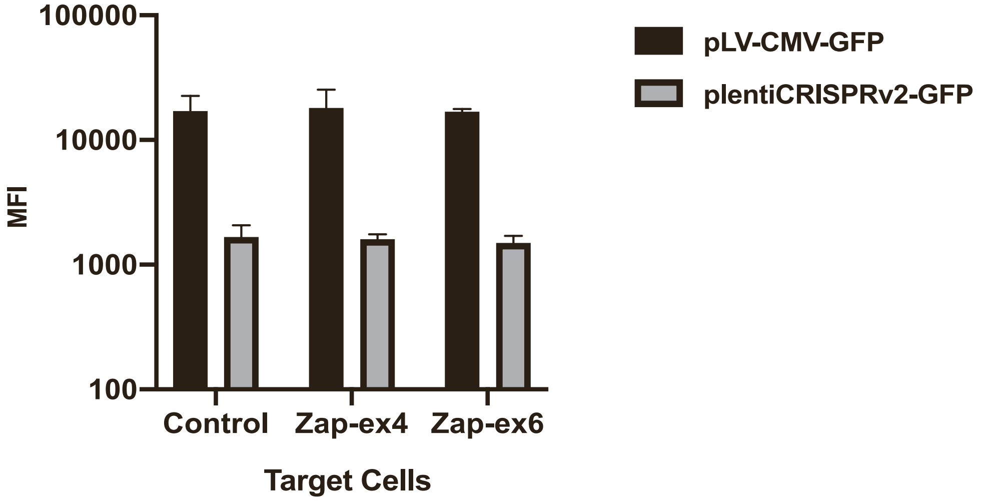

Figure S4. ZAP depletion does not affect the expression of transgenes under the control of CMV or EF-1a promoters.

(A) Media from HEK293T producer cells (CRISPR control, ZAP-ex4 or ZAP-ex6) transfected with pCMVΔR8.91, pVSV-G and the either pLV-CMV-GFP or plentiCRISPRv2-GFP, was used to transduce HEK293T CRISPR control target cells. **(B)** Media from HEK293T CRISPR control producer cells transfected with pCMVΔR8.91, pVSV-G and the either pLV-CMV-GFP or plentiCRISPRv2-GFP was used to transduce target cells (HEK293T CRISPR control, ZAP-ex4 or ZAP-ex6 cells). pLV-CMV-GFP contains the interval CMV promoter and plentiCRISPRv2-GFP contains the internal EF-1α promoter. MFI was determined by flow cytometry for GFP-positive target cells. The bar charts show the average values of six independent experiments. Data are shown as mean ± SD.

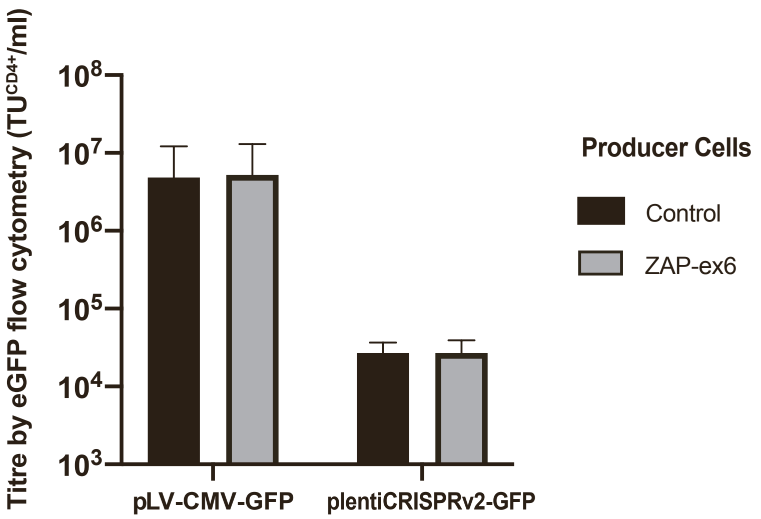

Figure S5. ZAP depletion in HEK293T producer cells does not increase vector titre in target primary human CD4+ T cells

Media from HEK293T CRISPR control and ZAP-ex6 producer cells transfected with pCMVΔR8.91, pVSV-G and pLV or plentiCRISPRv2-GFP was used to transduce target primary human CD4+ T cells. Infectious titres were determined by flow cytometry of GFP-positive target cells. The bar charts show the average values from four independent donors. Data are shown as mean ± SD.

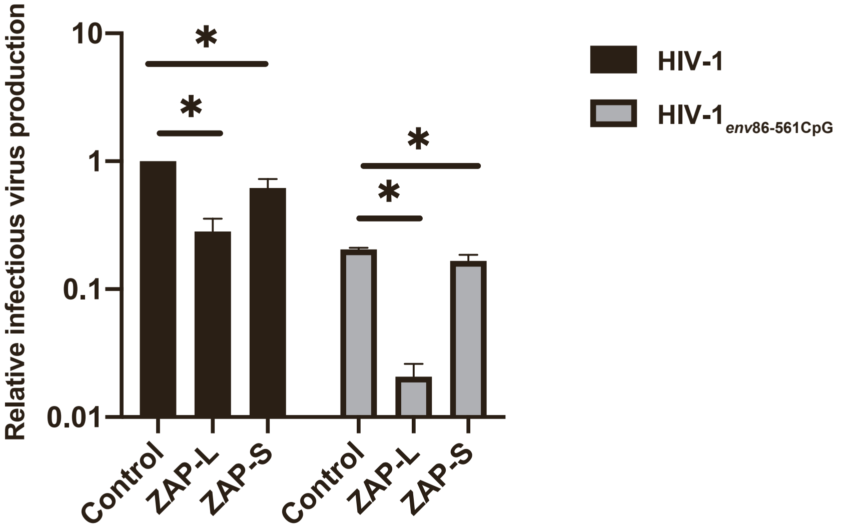

Figure S6. ZAP overexpression inhibits both wild type HIV-1 and HIV-1$_{env86\text{-}561CpG}$.

HEK293T cells were transfected with wild type pHIV-1 or pHIV-1$_{env86\text{-}561CpG}$ and either Control (pcDNA3), pZAP-L or pZAP-S. The culture supernatants were used to infect TZM-bl reporter cells to measure infectious virus production. The bar chart shows the average values of three independent experiments. Data are shown as mean ± SD, *$p<0.05$ as determined by an unpaired *t*-test.

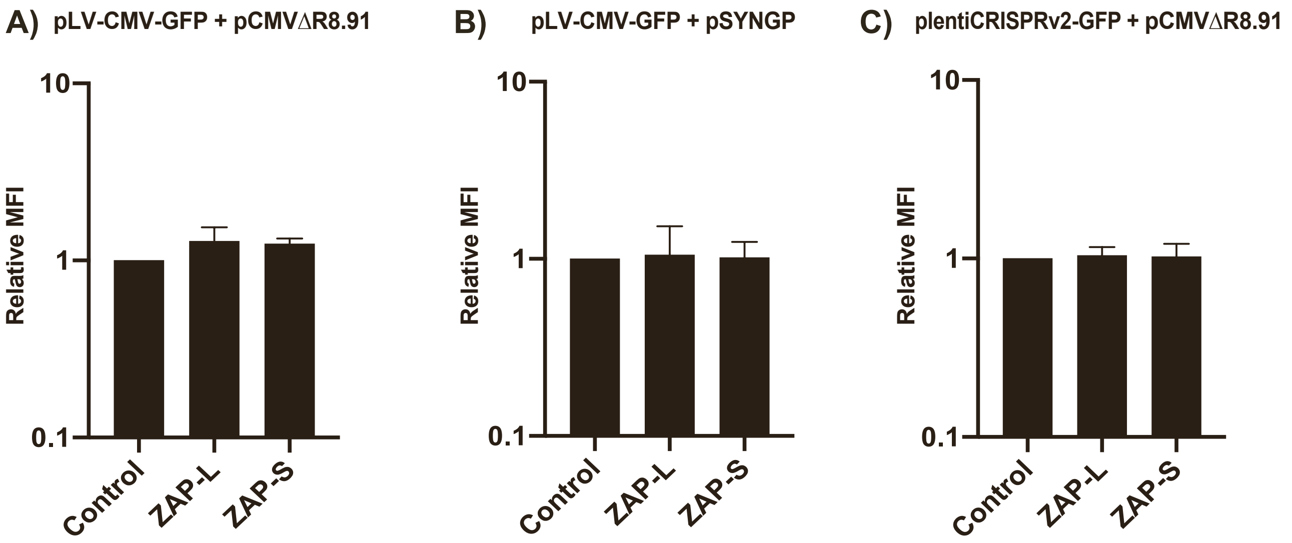

Figure S7. ZAP overexpression does not affect transgene expression in a lentiviral vector.

Media from HEK293T cells transfected with the indicated genome plasmid and packaging vector plus pVSV-G and either pcDNA4, pZAP-L or pZAP-S was used to transduce HEK293T target cells. MFI was determined by flow cytometry for GFP-positive target cells. The bar charts show the average values of three independent experiments. Data are shown as mean ± SD.

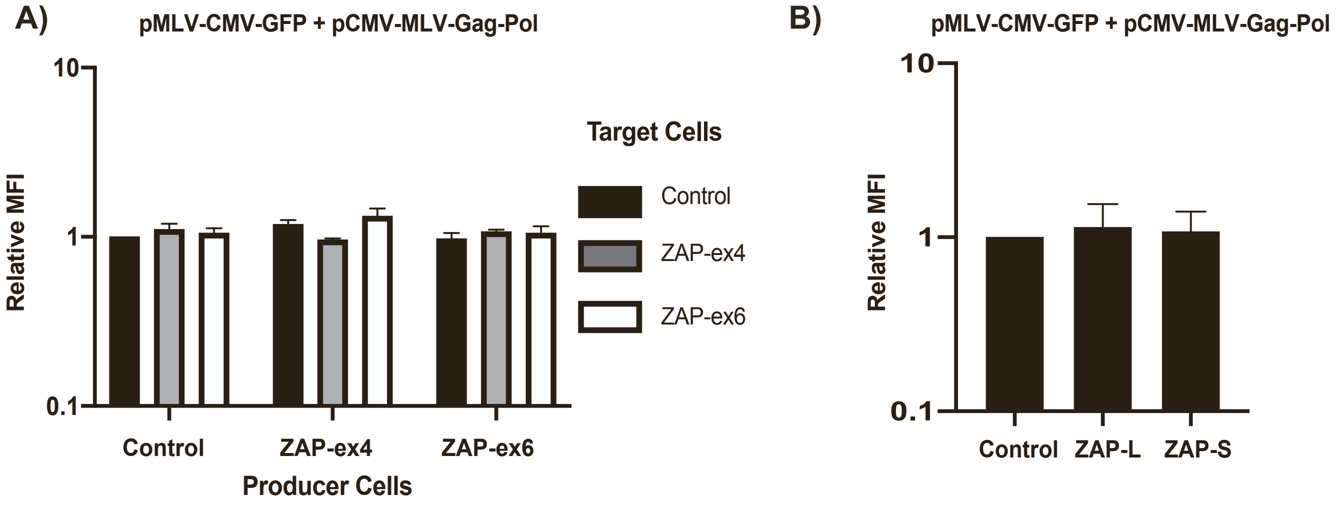

Figure S8. ZAP depletion or overexpression does not affect transgene expression in a gammaretroviral vector.

(A) Media from CRISPR control, ZAP-ex4 or ZAP-ex6 producer cells transfected with pMLV-CMV-GFP, pCMV-MLV-Gag-Pol and pVSV-G was used to transduce target cells (HEK293T CRISPR control, ZAP-ex4 or ZAP-ex6 cells). **(B)** Media from HEK293T cells transfected with pMLV-CMV-GFP, pCMV-MLV-Gag-Pol, pVSV-G and either pcDNA4, pZAP-L or pZAP-S was used to transduce HEK293T target cells. MFI was determined by flow cytometry for GFP-positive target cells.The bar charts show the average values of three independent experiments. Data are shown as mean ± SD.
